# Spatial distribution, movements, and geographic range of Steller sea lions (*Eumetopias jubatus*) in Alaska

**DOI:** 10.1371/journal.pone.0208093

**Published:** 2018-12-26

**Authors:** Lauri A. Jemison, Grey W. Pendleton, Kelly K. Hastings, John M. Maniscalco, Lowell W. Fritz

**Affiliations:** 1 Division of Wildlife Conservation, Alaska Department of Fish and Game, Douglas, Alaska, United States of America; 2 Department of Science, Alaska SeaLife Center, Seward, Alaska, United States of America; 3 Marine Mammal Laboratory, National Marine Fisheries Service, Seattle, Washington, United States of America; University of Sydney, AUSTRALIA

## Abstract

The two stocks of Steller sea lions (*Eumetopias jubatus)* in Alaska include an endangered western stock, recently recovering in parts of its range following decades of decline, and an eastern stock which was removed from the U.S. Endangered Species List in 2013 following increasing numbers since the 1970s. Information on overlapping distributions of eastern and western sea lions is needed for management considerations. We analyzed >30,000 sightings collected from 2000–2014 of 2,385 sea lions that were branded as pups at 10 Alaskan rookeries to examine mesoscale (mostly <500km) spatial distribution, geographic range, and geographic population structure based on natal rookery, sex, and age during breeding and non-breeding seasons. Analyses of summary movement measures (e.g., natal rookery, sex, and age-class differences in spatial distribution and geographic range) indicate wide variation in rookery-specific movement patterns. Correlations between movement measures and population dynamics suggested movement patterns could be a function of density dependence. Animals from larger rookeries, and rookeries with slower population growth and lower survival, had wider dispersion than animals from smaller rookeries, or rookeries with high growth and survival. Sea lions from the largest rookery, Forrester Island, where survival and population trends are lowest, were the most widely distributed. Analysis of geographic population structure indicated that animals born in the eastern Aleutian Islands had the most distinct movements and had little overlap with other western sea lions. Northern Southeast Alaska, within the eastern stock, is the area of greatest overlap between stocks, and is important to western animals, especially those born in Prince William Sound. Detailed knowledge of distribution and movements of western sea lions is useful for defining recovery and population trend analysis regions that better reflect dispersion and population structure and provides valuable information to managers as critical habitat is re-evaluated and the location of the stock boundary reconsidered.

## Introduction

Knowledge of the spatial and temporal distribution of marine vertebrates is necessary for understanding their basic ecology, and for habitat protection and species conservation. Many factors can influence a species’ distribution including prey availability, predator avoidance, and the need for adequate birthing sites. On an individual level, sex, age, and reproductive status affect an animal’s movement patterns and distribution.

Movements within a species’ range are undertaken for a variety of reasons that may vary over multiple timescales and among individuals. For example, multi-day movements occur during foraging trips by central place foragers (animals that require round-trips between foraging areas and a home base), such as Steller sea lions (SSL; *Eumetopias jubatus*) [1] and northern fur seals (*Callorhinus ursinus*) [2]. Seasonal migrations between breeding grounds and wintering sites are well known in a number of marine vertebrates, including Arctic terns (*Sterna paradisaea*); [3], gray whales (*Eschrichtius robustus*); [4; 5] and humpback whales (*Megaptera novaeangliae*); [6; 7]. Multi-year movement patterns also exist, such as for loggerhead sea turtles (*Caretta caretta*) that migrate to natal nesting beaches on average every 2.5–3 years from their home feeding ground, where distances between nesting beaches and feeding grounds can vary from <100km to >2000km [8–10].

Natural and anthropogenic changes to the environment can influence habitat quality and behavior of prey and predators, and therefore the distribution and movements of marine animals [11–13]. In polar regions, sea ice quality, quantity, as well as its seasonal advance and retreat, influence the distribution of pagophilic pinnipeds [14–16]. Ringed seals (*Phoca hispida*) and walruses (*Odobenus rosmarus*) prefer areas of extensive sea ice coverage, where adult ringed seals excavate pupping lairs for protection from weather and predators [14; 15], and walruses have adequate substrate to rest and nurse their young [15]. Anthropogenic sound associated with activities such as oil and gas development and naval military activities can disrupt movement patterns of cetaceans and can result in strandings [17–19]. The presence and activity of boats during commercial wildlife viewing can change behavior, for example by causing harbor seals on ice flows to enter the water, a particular concern for young pups [20] or by reducing the time whales spend feeding and resting [21]. Commercial fishing operations can alter marine mammal behavior in a variety of ways, including through localized prey depletions, interactions resulting in entanglements in or ingestion of gear, or depredation of catch [22–24]. Knowledge of a species’ distribution is necessary for understanding potential effects of both anthropogenic activities and naturally occurring changes in the environment, allowing for more successful management and conservation.

SSLs occur around the North Pacific rim from central California to northern Japan [25]; haulouts (where animals rest on shore, but few pups are born) and rookeries (where males establish territories and most breeding and births occur) throughout this range are numerous, geographically widespread, and well documented during the breeding season [26–29]. Few studies have examined how SSLs born at different rookeries are distributed throughout the species’ range during the breeding season, with even fewer studies during the non-breeding season [30–33]. Adult male SSLs arrive at rookery sites in early-mid May to establish territories; females arrive shortly after the males with parturition occurring between mid-May and mid-July [34; 35]. Females usually give birth for the first time at ages 5–7 [35; 36] and exhibit moderately high (0.776–0.859) natal philopatry (first breeding occurs at the natal site) [30; 37] and in Southeast Alaska (SEAK), very high breeding philopatry [37]. Most pups are weaned around age 1, but some pups continue to nurse until 2 or 3 years of age [38; 39] or in rare cases, beyond 3 years [35]. Male SSLs are reproductively mature (produce sperm) by ages ~5–7 [36; 40] but generally are not socially mature nor of sufficient size to hold a territory until age ~9+ [35; 40; 41]. In SEAK, the proportion of males holding territories at their natal site varied by birth location, but overall was moderately high [41].

**Fig 1:**
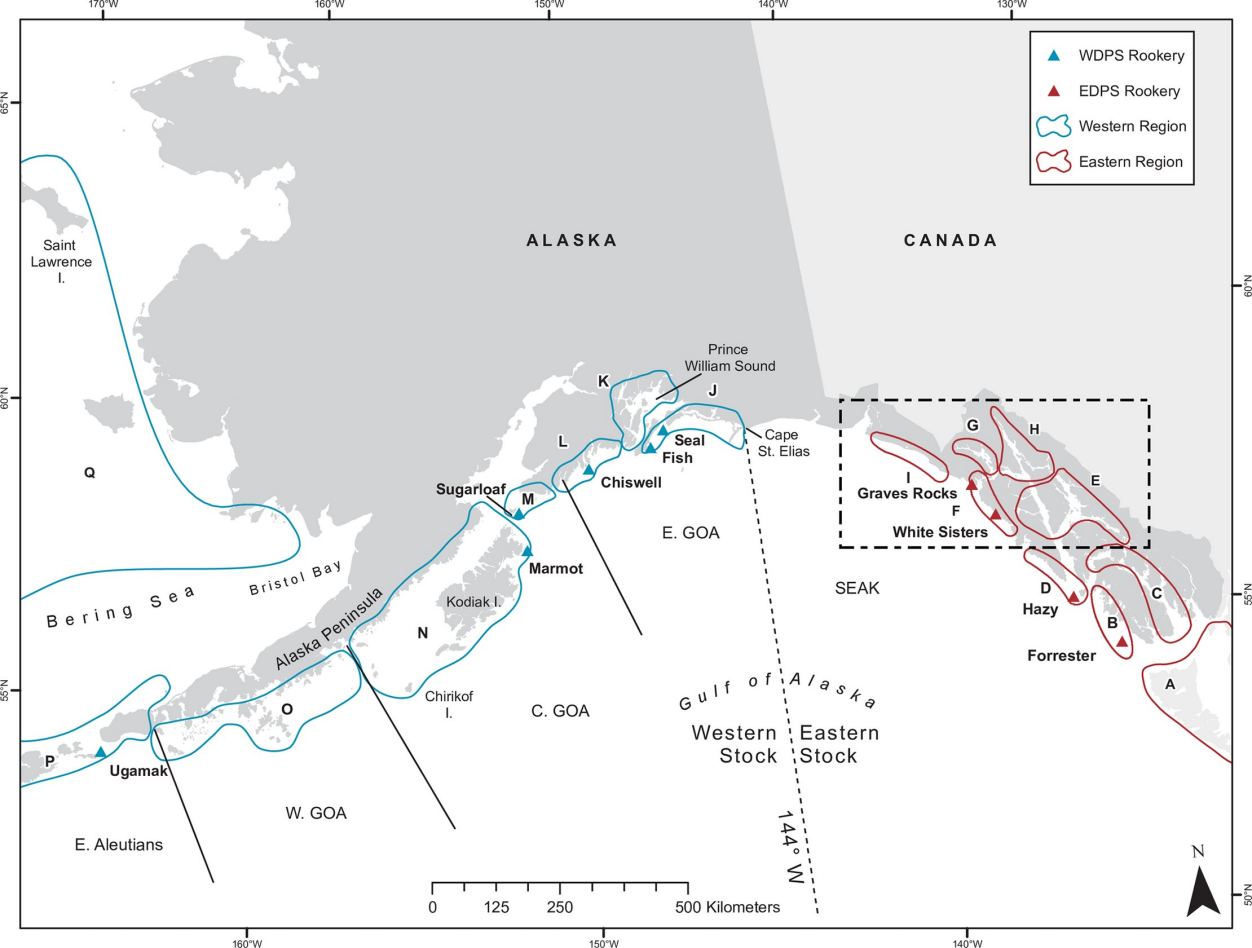
Rookeries where Steller sea lions were branded and regions where brand-resight surveys were conducted. The primary study area, from Chirikof Island to Forrester Island, where Steller sea lions in Alaska were branded at natal rookeries in 2000–2011 and resighted from 2000–2014. Regions (circled and lettered) represent areas used to analyze mesoscale movements and distribution. Solid black lines indicate National Marine Fisheries Service-designated sub-regions (SEAK = Southeast Alaska, E. GOA = eastern Gulf of Alaska, C. GOA = central Gulf of Alaska, W. GOA = western Gulf of Alaska, E. Aleutians = eastern Aleutian Islands). Dashed line identifies stock boundary division at 144°W. Black dashed box indicates area within the eastern stock with the highest resight density of western stock sea lions.

Understanding the spatial and temporal distribution of animals among haulouts and rookeries, based on birth location is of interest for conservation of SSLs in Alaska as population trends have varied regionally and over time [42]. SSL populations underwent steep declines in the central and western Gulf of Alaska and the Bering Sea from the late 1970s through the 1990s; the timing and rate of the decline varied among regions [42–44]. The decline led to the 1990 listing of SSLs as “threatened” under the U.S. Endangered Species Act [45]. Differences in population trends and genetics [46; 47] resulted in the classification of SSLs as two distinct population segments, or stocks, with the division at 144^o^W longitude. Eastern stock (or ‘eastern’) SSLs retained their “threatened” status whereas the listing for declining western stock (or ‘western’) sea lions was revised to “endangered” in 1997 [48]. Since the 1970s, the number of SSLs within the eastern stock (SEAK south through California; Fig 1) increased [29; 49], and in 2013, the eastern stock was removed from the U.S. Endangered Species List [50]. The number of SSLs within the western stock in Alaska reached their lowest overall abundance in the early 2000s and although they have increased at ~2%/yr since, there has been considerable regional variability in population trend including: increasing population trends in the Gulf of Alaska and eastern Aleutians since ~2003 and continuous decline in the central and western Aleutian Islands since the late 1970s [42].

The U.S. Marine Mammal Protection Act of 1972 provides protection for all marine mammals within the waters of the United States. Endangered western sea lions are afforded additional protections; for example, the National Marine Fisheries Service (NMFS) is required to designate critical habitat for species listed under the Endangered Species Act. Fishery management measures have been established in the western stock to spread out fishing effort over broad areas and to restrict fishing within some areas designated as critical habitat. Although proposed to be separate subspecies [51], it is not possible to visually distinguish between eastern and western stock sea lions unless an animal is permanently marked with a unique identifier. Based on marked individuals and genetic information, a substantial reproductive *mixing zone* exists between the two stocks within the eastern region, with evidence of permanent emigration of western females to the eastern stock and their reproduction at rookeries nearest the stock boundary [33; 52]. Movement probabilities across the stock boundary based on sex, age, and region of birth have been estimated using mark-recapture models of individually-marked SSLs [33]. Currently, individuals that are born in the western stock but feed, breed, or haul out in the eastern stock are not afforded the same protections as when they remain in the west. Thus, detailed information on how distribution, movement, and geographic range vary with natal rookery, sex, and age, are needed to better identify sites used by endangered western SSLs to better provide for their conservation. Associations between movement patterns and population processes may also provide insight into population response of SSLs to environmental variation, an important relationship which is currently poorly understood.

Studies that provide detailed information on a species' habitat use and movement patterns are often accomplished through the application of telemetry devices (tags), which have been used on marine mammals in the wild for more than 50 years [53; 54]. However, telemetry studies of SSLs, in which tags are usually secured to hair using epoxy, provide < 1 year of data due to molting [55]. We permanently marked SSLs as pups at their natal rookeries and gathered data with a geographically-broad resighting program, which provided movement and distribution information for SSLs from birth to 14 years of age.

Our study objectives were to:

1. Describe the mesoscale spatial distribution of SSLs during the breeding season by natal rookery, also accounting for sex and age-class.
2. Estimate average distances of SSLs from their natal rookery by sex and age during the breeding season, and maximum observed distance during any season by sex and age.
3. Quantify geographic ranges of SSLs by natal rookery, also accounting for sex and age-class, using data from the breeding and non-breeding seasons.
4. Examine geographic population structure based on shared site use by branded SSLs during the breeding and non-breeding seasons.
5. Compare regional movement and distribution patterns to regional population dynamics.

## Methods

This research, including procedures for animal capture, handling, marking, and resighting, was approved under permits issued by the NMFS to the Alaska Department of Fish and Game (ADF&G, Permit Numbers 358–1564, 358–1769, 358–1888, 14325, and 18537), the Alaska Fisheries Science Center—Marine Mammal Lab (Permit Numbers 782–1532, 782–1768, 782–1889, 14326, and 18528), and the Alaska SeaLife Center (Permit Numbers 881-1668-05, 881-1890-02, and 14324). This research was permitted annually by ADF&G, Marine Mammal Lab, and Alaska SeaLife Center Institutional Animal Care and Use Committees.

The ADF&G, the Marine Mammal Lab, and the Alaska SeaLife Center captured and permanently marked by hot-branding [56] samples of ~one to five week old SSL pups on their natal rookeries in Alaska during 2000–2011. Branding was performed under isoflurane gas anesthesia [57]. Branding of SSL pups in Alaska, including disturbance to the rookery, had little or no effect on survival of pups post-branding [58; 59]. In total, 4,385 pups were branded with unique alpha-numeric marks at 10 rookeries ranging from SEAK, the eastern and central Gulf of Alaska, and the eastern Aleutian Islands (Table 1, Fig 1). Resightings of all branded animals were included except for six animals whose sex was not recorded at the time of branding and who were not resighted as adults, and an additional nine animals whose sex was unknown due to a poor or misshapen brand that could not be matched with certainty to a specific individual. We included data of other SSLs with similarly poor brands that were seen as older animals and whose sex and natal rookery could be determined by process of elimination and by comparing their photographs to a master photograph library to ensure consistent identity over time. Mis-sexing during pup branding was rare: e.g., of 398 pups marked by the ADF&G and seen at >8 years old, only 9 (2.3%) had sex incorrectly assigned at branding.

**Table 1:** Number of Steller sea lions branded as pups at their natal rookery in eastern and western stocks within Alaska.

Stock	Natal Rookery	Branding years	Number branded	Pup count (year)	Number of pups^b^
East	Forrester	2001–04	995	2015	3954
East	Hazy	2001,03,05	539	2015	1994
East	White Sisters	2002,04,05	368	2015	910
East	Graves	2002,05	93	2015	502
	*East total*		*1995*		
West	Prince William Sound (Seal)	2001,03,05	255	2015	674
West	Prince William Sound (Fish)	2001	32	2015	340
West	Chiswell	2005,07,08,10	199	2015	102
West	Marmot	2000,02,04,08,10	434	2015	624
West	Sugarloaf	2000,02,04,08,10	559	2015	902
West	Ugamak	2001,03,05,09,11	911	2014	982
	*West total*		*2390*		
	**TOTALS**		**4385**^a^		

Branding years, pup count years, and number of pups counted at study-area rookeries during the most recent aerial surveys.

^a^ Fifteen animals not included in final analyses: 6 animals where sex was unknown at time of branding plus 9 animals with uncertain brands and unknown sex during resighting

^b^ [42]

We conducted annual brand-resighting surveys at haulouts and rookeries throughout Alaska and northern British Columbia during May through August 2000–2014, with greatest survey effort (i.e., site visits) occurring between mid-May and mid-July [33]. The majority of the brand-resighting data was collected within the “core study area” (between Chirikof Island in the western Gulf of Alaska and the Forrester Island complex in southern SEAK; Fig 1) during 2000–2014. For a small section of the core study area (Marmot Island west to Chirikof Island), and the regions westward, brand resight data were only available from 2000–2012. We only used observations of branded animals with an associated photograph, where identity was confirmed against a master photograph library containing all branded animals [33; 38; 60].

We surveyed every major haulout and rookery from northern British Columbia through the eastern Aleutian Islands at least once during each breeding season, as weather and logistics allowed. Most surveys were conducted either from a small skiff near the haulout or from shore (see [33] for details on survey methods). Extra survey effort (2–3 days) was extended to most rookeries (11 of 14) and larger haulouts in the core study area, particularly after 2004 when branded females were approaching reproductive age. Survey effort was not uniform across the SSL range because surveys in different parts of Alaska were conducted by personnel from different agencies, and because logistical constraints varied among regions. Weather and logistics precluded surveys at some sites each year, especially in the western Gulf of Alaska (Fig 1) and the Aleutian Islands (west of areas shown in Fig 1). Surveys in northern British Columbia (Region A, Fig 1) were not conducted during 2010–2012 and 2014 due to lack of funds or permits. SSL researchers in the Pacific Northwest, Canada, and Russia provided brand-resighting data from Oregon through British Columbia, and from Russia, ensuring resighting coverage of branded animals was adequate within the range of SSLs.

Although SSLs are, at times, pelagic [61], our study area included only near-shore zones (rookeries and haul-outs) where SSLs spend much of their time during the breeding season [25; 62] and where marked animals can be observed. Survey effort was much lower outside the breeding season (i.e., August-April), but surveys were conducted in some areas in most years (see Jemison et al. [33]). There has been consistently high year-round effort at the Chiswell Island rookery since 2000, and at nearby haulouts since 2001, where remote cameras have been used to monitor branded animals.

### Analyses

We defined two seasons for analyses: breeding (females: 25 May-31 July, males: 10 May-14 July) and non-breeding (females: 1 Aug-24 May, males: 15 July-9 May). The male breeding season is shifted earlier than the female season to reflect the earlier arrival of males at rookeries in May and the degradation of some prime season territories and departure from rookeries of some territorial bulls starting about mid-July. Analyses included data for individuals ~ >0.5 years old (after 31 December of the year in which they were born), reducing uneven resighting effort among rookeries in the year that pups were born and branded. Data from animals branded at Ugamak Island were only used in selected analyses (maximum distance, geographic range, and geographic population structure) due to limited resighting data available west of Ugamak Island.

#### Objective 1: Mesoscale spatial distribution during the breeding season

Based on Raum-Suryan et al. [55] and our preliminary visual examination of distribution by sex and age, we divided the core study area into 13 Regions (B-N, Fig 1) and assigned an additional four regions outside the core study area (A: British Columbia, California, Oregon, and Washington, O-Q: west and north of Kodiak Island, Fig 1; the full extent of Region A is not shown on Fig 1 - southern British Columbia south through California). Core study area regions included 4 inner and 4 outer coast regions in SEAK (B-I, Fig 1) and 5 areas from Cape St. Elias to Kodiak Island (J-N, Fig 1). We considered this mesoscale because it was a finer spatial scale than the 2 stock divisions and the 7 survey sub-regions within Alaska delineated by Fritz et al. [42] (Fig 1) but a coarser temporal and spatial scale than would be obtained through telemetry. We examined distributions based on natal rookery, sex, and age-class. Females were divided into two age classes: juveniles (1–3 yrs) and adults (4+ yrs; females are usually mature at ≥4 yrs [35; 36]). Males begin breeding at a later age and so were divided into three age classes: juveniles (1–4 yrs), sub-adults (5–8 yrs, when they are sexually mature but unable to hold territories), and adults (9+ yrs, when they are capable of maintaining a breeding territory) [35; 40; 41]. We combined animals branded at Prince William Sound rookeries (Seal Rocks and Fish Island) due to the small number of animals branded at Fish Island (n = 32) and similar spatial distributions of sightings for animals from these sites. For each rookery-sex-age class group, we calculated an index of resight density for each of the regions as the proportion of individuals seen at least once in that region during the breeding season relative to the total number of individuals resighted in that group in any region in either season. If an individual was seen in more than one region at that age-class, it was included in the indices for all regions where it was seen. We expect that basing the density index on the proportion of individuals seen at least once, rather than the number of times each individual was seen, largely accounts for uneven survey effort among regions. We only computed the density index for groups with ≥ 10 individuals seen for that group.

A simple index of natal rookery-age-sex diversity for each region was determined by adding the number of rookery-sex-age class groups that had a resight proportion ≥0.100 within the region.

#### Objective 2: Mean and maximum distance from natal rookeries

We used Google Earth to manually measure the distances between natal rookeries and all sites (individual haulout or rookery) where ≥ 1 branded SSL was resighted. Distances were measured as the shortest paths between sites that did not cross land. SSLs occasionally occur off-shore, but most observations of SSL are on the continental shelf and nearshore [55; 61] so we also restricted between-site measurements to paths over the continental shelf. Using these distances, we calculated mean (during the breeding season) and maximum (using data from both seasons) distances of SSLs from their natal rookery. We used the same natal rookery and sex groups as for analysis of spatial distribution, except ages were not grouped into classes, allowing us to examine more fine-grained age patterns. We could maintain annual ages in these particular analyses without the summaries becoming too complex for presentation, as would have been the case for some of our other analyses (e.g., distribution, geographic range). An individual at a site at a given age was used only once when calculating mean distances, irrespective of how many times the animal was seen at that site. Maximum distances were the largest distance from the natal rookery of any individual in that group. The average distance provided a general index of dispersion for SSLs from each rookery, and the maximum distance provided an index of potential movement.

#### Objective 3: Geographic range

For each age-class, sex, and natal rookery group, we created a list of all sites where marked SSLs were seen at least once during either the breeding or non-breeding season. Using these lists and the between-site distances previously described, we determined the minimum spanning tree and its length. The minimum spanning tree is the shortest cumulative path that connects all nodes in a network; in our case it is the set of between-site connections that connects all of the used sites with the smallest cumulative length. We use spanning trees as visual depictions of the total geographic range used by SSLs from each group and to illustrate overlap, or lack thereof, among groups. We used the length of the spanning tree as an index of the geographic range size. To reduce the effect of a few very-long-distance observations where only 1 marked SSL from a natal rookery was seen at a site, we also computed ranges (i.e., spanning trees) that were based on lists of sites where we observed >1 marked SSL. We determined ranges for each rookery by sex and age class and by sex with age classes combined.

The number of animals branded and subsequently resighted varied among sex and age-classes within rookeries (range n = 27–264). Because range sizes are cumulative across animals, we were concerned that larger sample sizes may produce larger range sizes. We examined the effect of sample size on our spanning tree estimates of geographic range and used this information to compute a "relative" range size to account for sample size (see S1 Text for description of methods).

#### Objective 4: Geographic population structure

To investigate SSL population structure based on observed movement patterns, we conducted a cluster analysis grouping sites based on the number of branded SSLs seen at each pair of sites using data from both seasons combined, which better represents overall ranges for SSLs from each rookery. For each pair of sites, we computed a similarity value, which we then transformed to a dissimilarity by subtracting the similarity from 1. As our similarity measure, we used a modification of the Jaccard Index [63] of max(n_b_/n_1_,n_b_/n_2_), where n_b_ is the number of individuals common to both sites, n_1_ is the number seen at site 1 and n_2_ is the number seen at site 2; the Jaccard Index is the number at both sites divided by the number at either site. Our modified version accounts for the asymmetry in similarity within the site-pairs caused by large differences in the number of animals seen per site (i.e., 1–286). Our data for estimating similarity are the number of individuals shared between lists of SSLs from different sites, consequently making similarity measures used with continuous measures (e.g., Euclidean distance) inappropriate. Once we had computed the index for all pairwise combinations of sites, we grouped sites with the following algorithm. The pair of sites with the lowest dissimilarity (i.e., most similar lists of observed SSLs) was grouped and their dissimilarity recorded. This process was repeated until all sites, or groups of sites, were combined into a single final group containing all sites. From these results we constructed a dendrogram using the R package dendextend [64; 65] to illustrate population structure based on shared sightings of marked SSLs.

#### Objective 5: Movement measures and regional population dynamics

We used Pearson correlations to investigate relationships between estimated rookery-specific population dynamics measures (i.e., population trend, pup production, and both juvenile and adult survival probabilities) to rookery- and age-specific movement measures (i.e., proportion in the natal region, geographic range size, and mean distance from natal rookery). Correlation analyses use 1 observation of each population dynamics and movement measure for each rookery with separate analyses by sex. Data for estimating population trends and pup production were from [66]; survival estimates were from [60; 67; 68]. See S2 Text for more detailed information on data and analyses used to estimate population trend.

## Results

We observed 2,385 individual branded SSLs (54% of those that were branded as pups at 10 Alaskan rookeries) at least once (range 1–144 sightings/animal) after age ~0.5 years. We recorded 30,518 photo-confirmed sightings of these animals from 2000–2014.

### Objective 1: Mesoscale spatial distribution during the breeding season

SSLs were more dispersed at younger ages than at older ages: both sexes were more likely to be seen within their natal region at older ages than at younger ages. The proportion of males seen within their natal region ranged from 0.089–0.603 and 0.378–1.00 at ages 1–4 and 5+, respectively; for females, these ranges were 0.138–0.704 and 0.725–1.00 at ages 1–3 and 4+, respectively (Table 2). Female distribution, in terms of regions used, was similar at younger and older ages whereas for males, their distribution contracted at ages 5–8 and especially at age 9+ (S1–S10 Figs).

**Table 2:** Proportions of individual sea lions from each natal rookery, sex, and age-class seen in each Region during breeding season. The natal rookery-age-sex diversity index for each region is shown at bottom of table, in italics. In sex-age column, F = female, M = male and associated numbers are animal ages in years. Bold face, underlined proportions indicate natal region. Regions (A-Q) and NMFS-designated sub-areas are shown on Fig 1.

		Region																
		A	B	C	D	E	F	G	H	I	J	K	L	M	N	O	P	Q
Sex-age	Natal rookery	CA-BC	SEAK								EGOA			CGOA		WGOA	EAI	Bering
F1-3	Forrester	0.136	**0.366**	0.132	0.226	0.162	0.098	0.021	0.017	0.009								
	Hazy	0.022	0.118	0.029	**0.294**	0.419	0.132	0.022	0.103	0.007								
	White Sisters				0.094	0.160	**0.547**	0.189	0.075			0.009						
	Graves				0.037	0.037	**0.704**	0.296										
	Prince William Sound						0.226	0.081		0.032	**0.226**	0.194						
	Chiswell										0.059	0.353	**0.485**	0.015				
	Sugarloaf						0.031	0.015		0.008	0.123	0.069	0.162	**0.138**	0.185			
	Marmot				0.008		0.060	0.038			0.060	0.008	0.023	0.008	**0.602**			
F4+	Forrester	0.090	**0.846**	0.077	0.231	0.064	0.083	0.026	0.019									
	Hazy		0.146	0.012	**0.890**	0.451	0.122	0.037	0.134									
	White Sisters		0.043		0.071	0.114	**0.900**	0.286	0.143			0.014						
	Graves				0.045		**1.000**	0.500										
	Prince William Sound						0.175	0.070			**0.860**	0.298	0.035					
	Chiswell										0.057	0.257	**0.829**	0.029				
	Sugarloaf						0.033				0.198	0.066	0.132	**0.725**	0.275			
	Marmot						0.079	0.063			0.175	0.032	0.079	0.175	**0.810**	0.016		
M1-4	Forrester	0.090	**0.214**	0.102	0.083	0.169	0.124	0.071	0.071		0.034	0.023	0.026	0.008	0.053		0.008	0.004
	Hazy	0.007	0.059	0.026	**0.118**	0.388	0.191	0.118	0.164		0.026	0.033	0.020		0.046		0.007	
	White Sisters	0.007	0.037	0.007	0.037	0.096	**0.326**	0.274	0.259	0.007	0.022	0.007	0.022		0.022		0.007	
	Graves					0.111	**0.361**	0.500	0.194						0.028			0.028
	Prince William Sound	0.013	0.013			0.013	0.076	0.063	0.025		**0.190**	0.342	0.076		0.038	0.013		
	Chiswell						0.040	0.053			0.053	0.133	**0.493**		0.067	0.013		
	Sugarloaf				0.006	0.018	0.071	0.030	0.006		0.095	0.195	0.148	**0.089**	0.308	0.012	0.012	0.041
	Marmot	0.008	0.008		0.008	0.008	0.048	0.024			0.079	0.032	0.103	0.048	**0.603**		0.024	0.008
M5-8	Forrester	0.150	**0.589**	0.037	0.112	0.178	0.262	0.047	0.028		0.047	0.009			0.019		0.009	0.009
	Hazy		0.047	0.016	**0.453**	0.313	0.375	0.109	0.172		0.078	0.016	0.031		0.031			0.016
	White Sisters				0.015	0.090	**0.881**	0.209	0.194		0.045	0.060						
	Graves						**1.000**	0.379	0.069		0.069				0.034			0.034
	Prince William Sound						0.108				**0.649**	0.405	0.027	0.054	0.054			
	Chiswell												**0.895**	0.053				
	Sugarloaf						0.057	0.019			0.038	0.075	0.170	**0.396**	0.302	0.019	0.019	0.151
	Marmot					0.032	0.032				0.065	0.032	0.129	0.032	**0.710**		0.032	0.161
M9+	Forrester	0.053	**0.667**	0.053	0.105	0.105	0.228				0.018		0.018					
	Hazy		0.081		**0.378**	0.270	0.297	0.081	0.189									
	White Sisters						**0.927**	0.049	0.122			0.024						
	Graves						**0.955**	0.273	0.091									0.045
	Prince William Sound						0.095				**0.905**	0.238						
	Sugarloaf						0.037	0.037			0.074	0.037		**0.407**	0.074			0.111
	Marmot						0.143						0.071	0.071	**0.643**		0.071	
	*DIVERSITY*	*2*	*7*	*2*	*9*	*12*	*22*	*11*	*10*	*0*	*8*	*9*	*10*	*6*	*9*	*0*	*0*	*3*

Row totals do not add to 1 because an individual could be seen in more than one area (sum could be >1) and the denominator is the number of individuals from each rookery in each age class seen in either breeding or non-breeding seasons in any region. Proportions were calculated when n≥10 individuals. Data from Ugamak Island-branded SSLs are not included in the table due to incomplete observation histories west of Ugamak Island.

Of SSLs born in the eastern stock, animals born at Forrester and Hazy Islands were more widely distributed outside their natal and adjacent regions than those born at northern SEAK rookeries, especially in the younger age groups (Table 2, S1–S5 Figs). Forrester- and Hazy-born females were seen throughout SEAK and in British Columbia (mainly Forrester animals) but were never seen west of the stock boundary. White Sisters and Graves females were rarely (0.043) or never, respectively, seen in southern SEAK or farther south (Regions A-C); only one female from White Sisters was observed in the west during the breeding season, first as a juvenile, and later as an adult nursing a juvenile (Table 2). Graves Rocks females in particular had a narrow distribution with the proportion in their natal region 0.704 and 1.00 at ages 1–3 and 4+, respectively (Table 2). Males born in the eastern stock, except those born at Graves Rocks, were distributed throughout much of Alaska including the Bering Sea, and south into Canada and the Pacific Northwest (Table 2, S1–S5 Figs). Hazy Island males in particular had low proportions (0.118, 0.453 and 0.378) of individuals within their natal region at ages 1–4, 5–8, and 9+, respectively, relative to males born at other rookeries in the eastern stock (Table 2). Graves Rocks males, similar to females born at this site, were primarily concentrated in northern SEAK and were never seen in southern SEAK or farther south. A small proportion (0.028–0.045) of males from Graves Rocks did travel west, one traveling as far as northern Bristol Bay in the Bering Sea (Table 2).

Of SSLs born in the western stock, juvenile males were broadly distributed to the east and west of their natal region, whereas females from Marmot, Prince William Sound, and Chiswell rookeries were rarely seen west of their natal region (proportions ranged from 0–0.035, Table 2, S6–S10 Figs). Male and female SSLs born at Sugarloaf Island had the lowest proportion of individuals within their natal region at all ages (males: 0.089–0.407; females: 0.138–0.725) relative to animals born at other rookeries in the western stock (Table 2). Chiswell animals were less dispersed than other western sea lions; Chiswell females occupied their natal region or the nearest neighboring regions (Table 2, S6–S10 Figs). In general, Prince William Sound females either occupied their natal region (outer Prince William Sound east to the stock boundary; proportions 0.226–0.860), the adjacent region to the north (inner Prince William Sound; proportions 0.194–0.298) or moved to sites in northern SEAK (proportions 0.032–0.226), in their non-natal stock (Table 2, S6–S10 Figs).

Region F, which falls within the mixing zone area of northern SEAK, had the highest diversity index compared to all other regions (~2-10x greater than other regions, Table 2). Female and male SSLs marked at all rookeries in the core study area utilized this region during the breeding season, with the exception of Chiswell-born females. A lower proportion of Forester and Hazy females were present in Region F (0.083–0.132) compared to Prince William Sound females (0.175–0.226), despite the closer proximity of Forester and Hazy Islands to this Region (Fig 1, Table 2). Region F rookeries (Graves Rocks and White Sisters) are the only known sites where western females have moved from their natal stock to the opposite stock to give birth [33]. Region F includes two rookeries, possibly contributing to a higher diversity index. However, even if we combined other regions with neighboring rookeries (e.g., Regions L and M, or Regions B and D), Region F’s diversity index would still be ~1.5-2x greater than those combined regions. For regions that do not contain a rookery, those in closest proximity to Region F had the highest diversity indexes (Regions E, G, and H). Distribution patterns of western animals during the breeding season suggest areas in SEAK where greater protection may assist conservation of the endangered stock: (see black box in Fig 1) western adults and juveniles of both sexes, including breeding females, occurred regularly in Regions F and G, and to a lesser extent Region I (S1–S10 Figs). Western males also used Region E (Frederick Sound-Chatham Strait), and Prince William Sound males used Region H (Lynn Canal).

### Objective 2: Mean and maximum distance from natal rookeries

**Fig 2:**
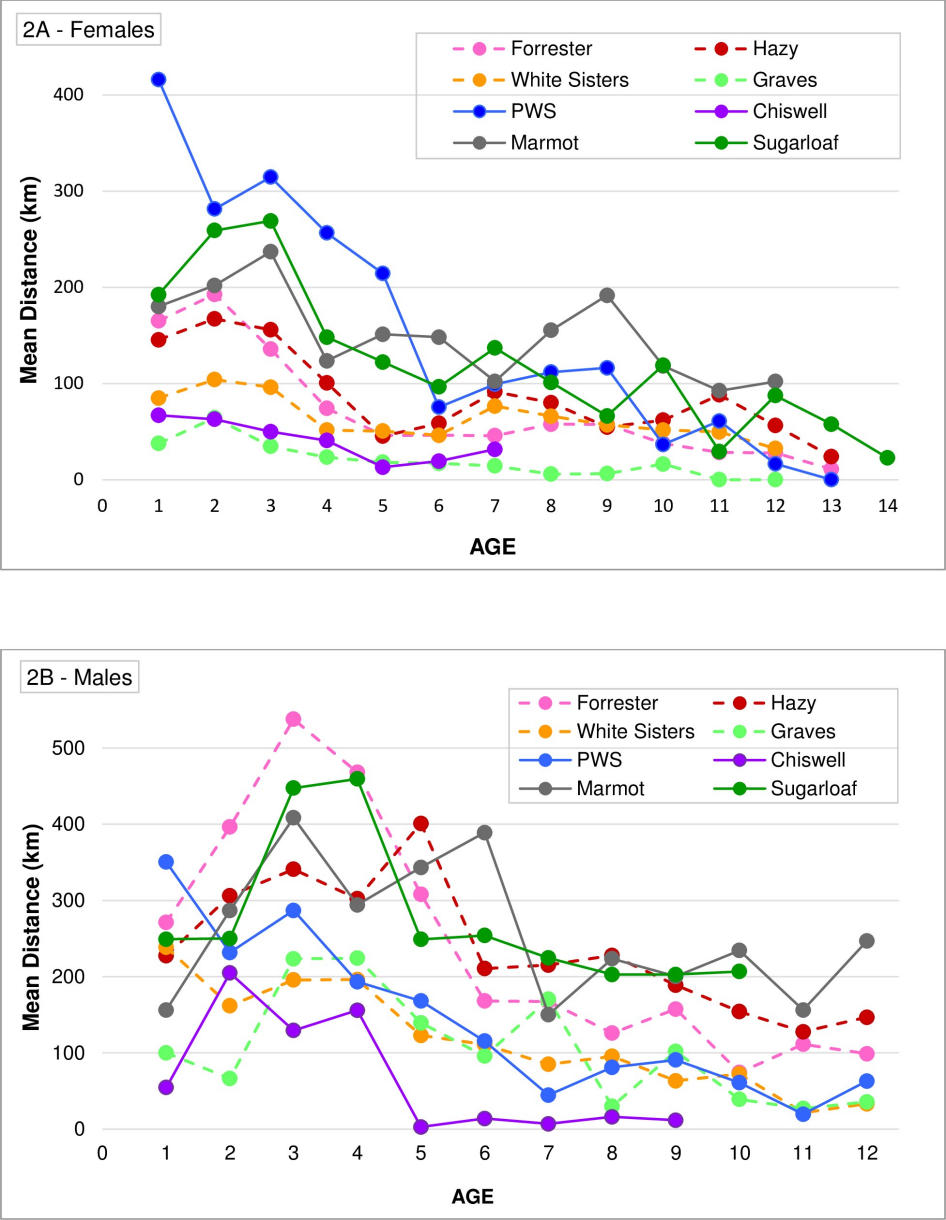
Mean distances Steller sea lions were observed from their natal rookery in Alaska. (A) Female and (B) male distances were based on age and rookery of origin during the breeding season. Distances were plotted for each age group where ≥5 animals were observed. Dashed lines represent eastern stock rookeries and solid lines represent western stock rookeries.

During the breeding season, SSLs were resighted at greater distances, on average, from their natal rookeries at younger ages than older ages (Fig 2; see S1 Table for confidence intervals on mean distances). With the exception of Prince William Sound-born animals, males >1 year were nearly always farther from their natal rookery than females of similar ages (Fig 2). Average female distance from natal rookery declined markedly around breeding age (~4–6); for males, this distance declined around age 7 (Fig 2). Overall, eastern and Chiswell females remained nearer their natal rookery than females from Prince William Sound, Marmot, and Sugarloaf (Fig 2). Females from Chiswell and Graves Rocks moved little at any age, on average remaining within <70km of their birth site as juveniles and ~40km as adults. By contrast, females from Prince William Sound averaged ~200-400km from their natal rookery until age 6; these females were farther from their natal rookeries than males from all rookeries at age 1, and farther than Prince William Sound males through age 5 (Fig 2, S1 Table). Males born at Sugarloaf, Marmot, Forrester, and Hazy Islands tended to be farther from their natal rookery compared with other males (Fig 2).

**Fig 3:**
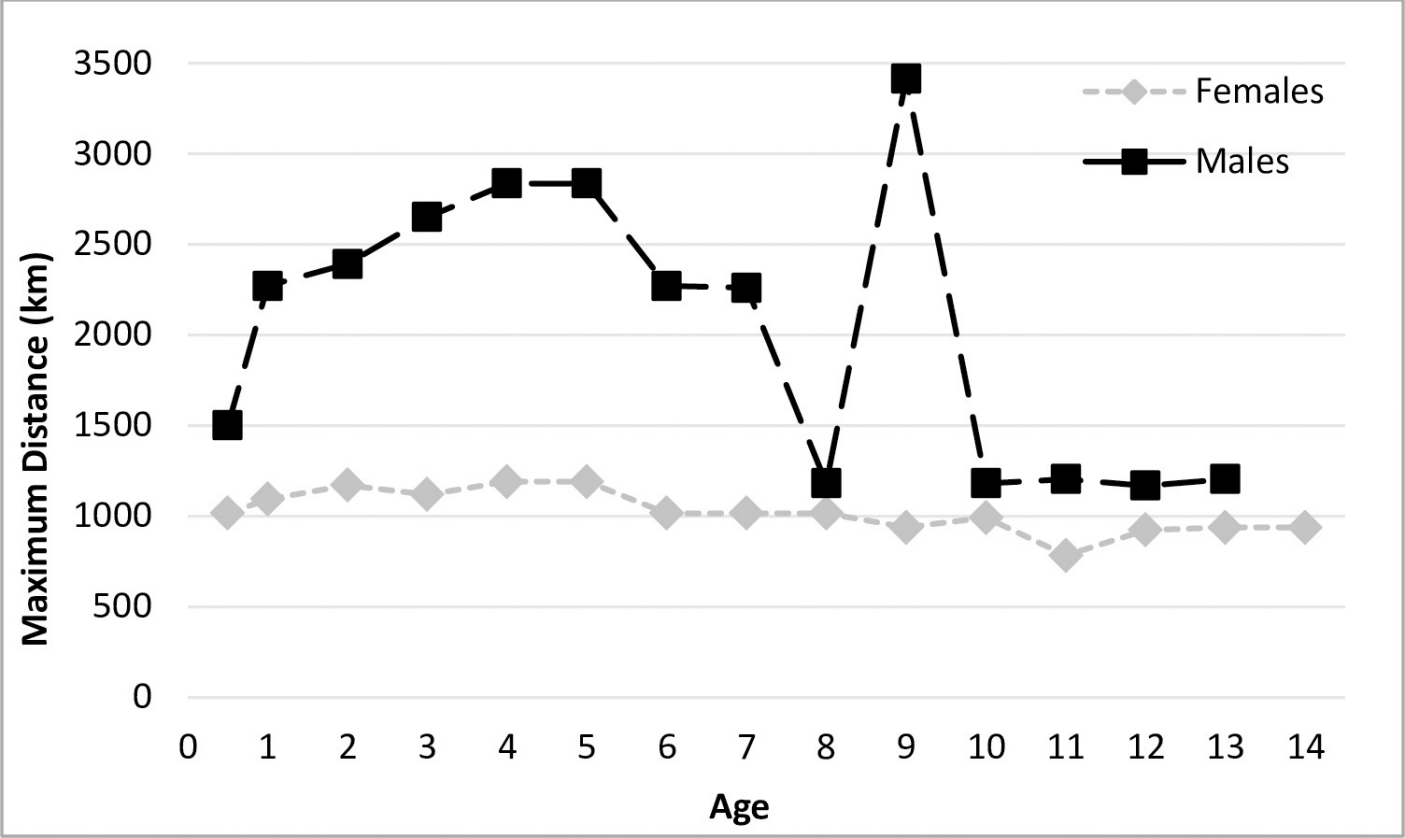
Maximum distances Steller sea lions were observed from their natal rookery in Alaska. Distances were based on data collected during the breeding and non-breeding seasons.

Based on maximum distances observed, within the first year of their life SSL females are capable of traveling 1000km from their natal rookery, and this maximum distance from the natal rookery varies little with age for females (Fig 3). Maximum distances males traveled from their natal rookery were about twice as high from 1–7 yrs (~2400km) compared with SSLs 8+ yrs, when maximum distances were more similar to females (~1200km), with the exception of a few very long-distance movements. A 13 month old male was resighted ~2,300km from his natal rookery (Forrester Island to Ugamak Island); older males have been documented at nearly 3,500km from their natal rookery (Fig 3).

### Objective 3: Geographic population range

Geographic ranges differed most by natal rookery and sex, with less pronounced differences among age classes. Males had larger ranges than females for all rookeries, often 1.5–3 times larger (Table 3). SSLs born at Graves Rocks and Chiswell Island (especially females) had smallest ranges (females, all ages: ~500km; males, all ages: 654km and 1232km for Graves and Chiswell, respectively). Female SSLs born at Forrester, Sugarloaf, and Marmot Islands had the largest ranges (2011km, 1941km, and 1879km, respectively) and males born at Forrester (6028km) and Sugarloaf (4911km) had the largest ranges (Table 3). SSLs from other sites had intermediate-sized ranges and range size declined with age class for males but not females (Table 3). Based on the random subset analyses, geographic range estimates increased with increasing sample size of individuals included in the analysis (see S1 Text and Fig A in S1 Text for results of this analysis).

**Table 3:** Geographic ranges (i.e., minimum spanning tree lengths [km]) based on locations used by Steller sea lions, by natal rookery, sex, age class, and all ages combined. Values are the distances among sites that were used by 2 or more marked SSLs; the values in parentheses include sites with only a single marked sea lion seen. Sample sizes for F:M are shown below rookery name.

Rookeries	Female				Male				
n = F/M	Age				Age				
	1–3	>4	All ages	Relative range ^a^	1–4	5–8	9+	All ages	Relative range ^a^
				(95% CI)					(95% CI)
Forrester	1773	1820	2011		4470	2412	1456	6028	
247/264	(2133)	(1920)	(2243)		(7482)	(5472)	(5560)	(9162)	
Hazy	1266	1300	1653	0.90	2981	2013	958	3073	0.66
133/146	(2525)	(1774)	(2604)	(0.76, 1.03)	(6308)	(4062)	(4063)	(7458)	(0.49, 0.84)
White Sisters	793	1088	1187	0.68	1982	1567	1119	3136	0.75
104/136	(1708)	(1912)	(2092)	(0.54, 0.81)	(4124)	(4215)	(1396)	(4969)	(0.53, 0.98)
Graves	270	420	494	0.55	454	228	380	654	0.41
27/37	(1458)	(900)	(1753)	(0.24, 0.86)	(3307)	(3059)	(2569)	(3542)	(0.05, 0.77)
PWS ^b^	1039	950	1051	0.66	1695	1197	903	1767	0.48
71/80	(1308)	(1355)	(1367)	(0.48, 0.83)	(4186)	(1765)	(1113)	(4247)	(0.34, 0.63)
Chiswell	493	297	533	0.33	1062	674		1232	0.34
70/71	(656)	(555)	(702)	(0.24, 0.42)	(2030)	(874)		(2053)	(0.24, 0.44)
Sugarloaf	1801	1710	1941		3863	3107	2465	4911	
147/170	(3131)	(2112)	(3140)		(7814)	(3406)	(3755)	(7895)	
Marmot	1872	1513	1879	1.02	3464	2210	937	3463	0.83
138/126	(2630)	(2973)	(3674)	(0.87, 1.17)	(5484)	(3158)	(2216)	(5491)	(0.58, 1.08)
Ugamak	1347	993	1582	0.85	1685	1979	1924	2590	0.62
117/134	(1909)	(1500)	(2022)	(0.73, 0.98)	(5517)	(4709)	(2270)	(5772)	(0.43, 0.81)

Spanning tree estimates are based on sites used at any time of year, including both breeding and non-breeding seasons. Relative ranges are the mean size relative to ranges of SSLs from Forrester and Sugarloaf Islands, the islands with the largest observed ranges, adjusted for sample size via simulation.

^a^ Geographic range relative to those from Forrester and Sugarloaf Islands, adjusted for the number of SSLs observed; see S1 Text.

^b^ PWS = Prince William Sound

**Fig 4:**
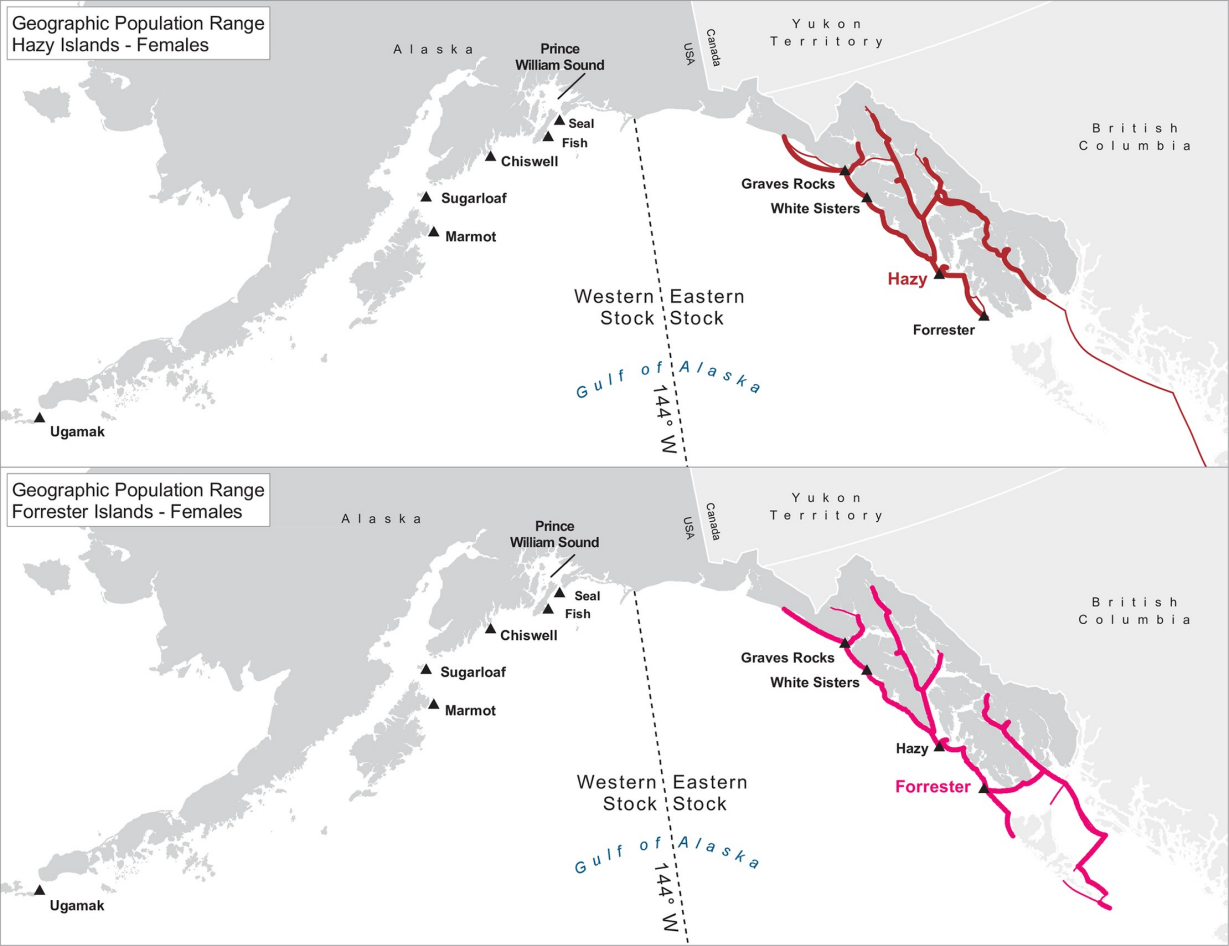
Geographic population range of female Steller sea lions born at Forrester and Hazy Islands. Population range of female Steller sea lions born at Forrester and Hazy Islands estimated using minimum spanning tree based on year-round observations of sea lions (of all ages combined) for each natal rookery. Thicker solid line indicates movement between two sites by >1 sea lion; thin lines indicate between-site movement by a single individual.

**Fig 5:**
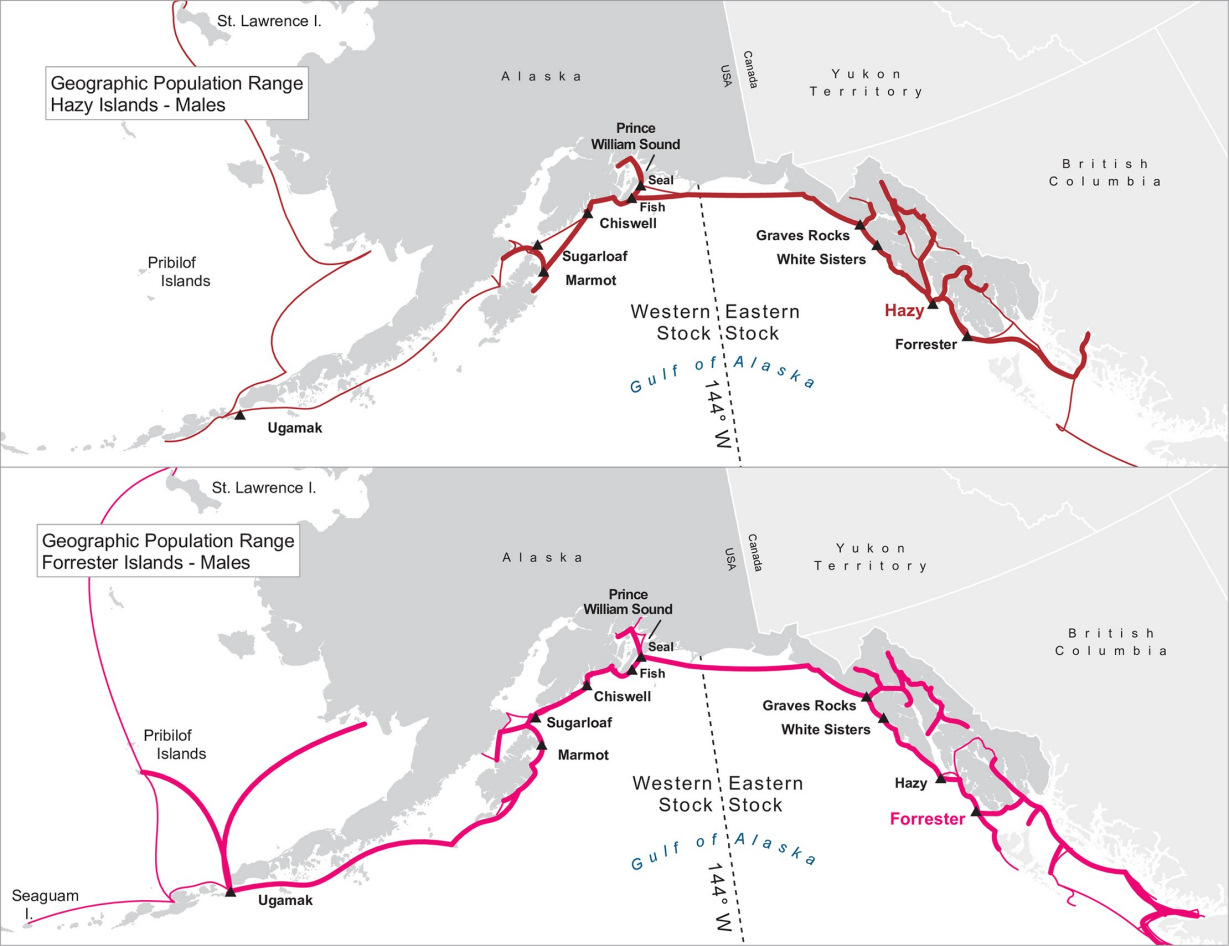
Geographic population range of male Steller sea lions born at Forrester and Hazy Islands. Population range of male Steller sea lions born at Forrester and Hazy Islands estimated using minimum spanning tree based on year-round observations of sea lions (of all ages combined) for each natal rookery. Thicker solid line indicates movement between two sites by >1 sea lion; thin lines indicate between-site movement by a single individual.

**Fig 6:**
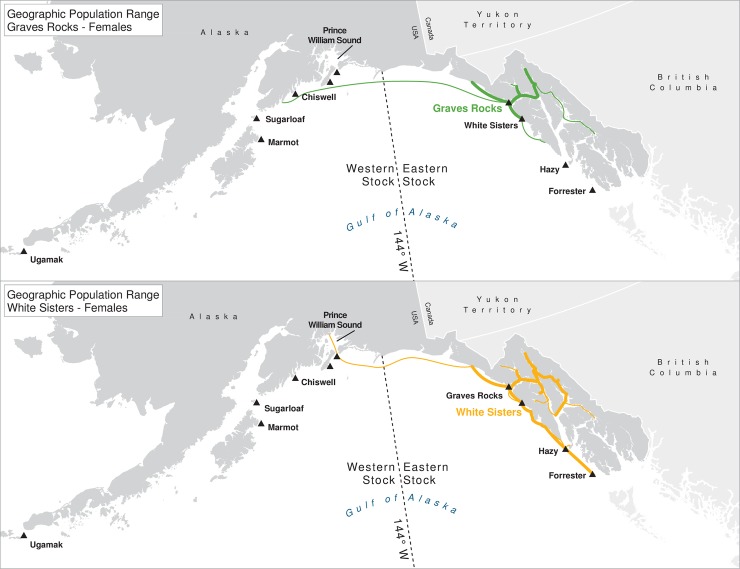
Geographic population range of female Steller sea lions born at Graves Rocks and White Sisters. Population range of female Steller sea lions born at Graves Rocks and White Sisters estimated using minimum spanning tree based on year-round observations of sea lions (of all ages combined) for each natal rookery. Thicker solid line indicates movement between two sites by >1 sea lion; thin lines indicate between-site movement by a single individual.

**Fig 7:**
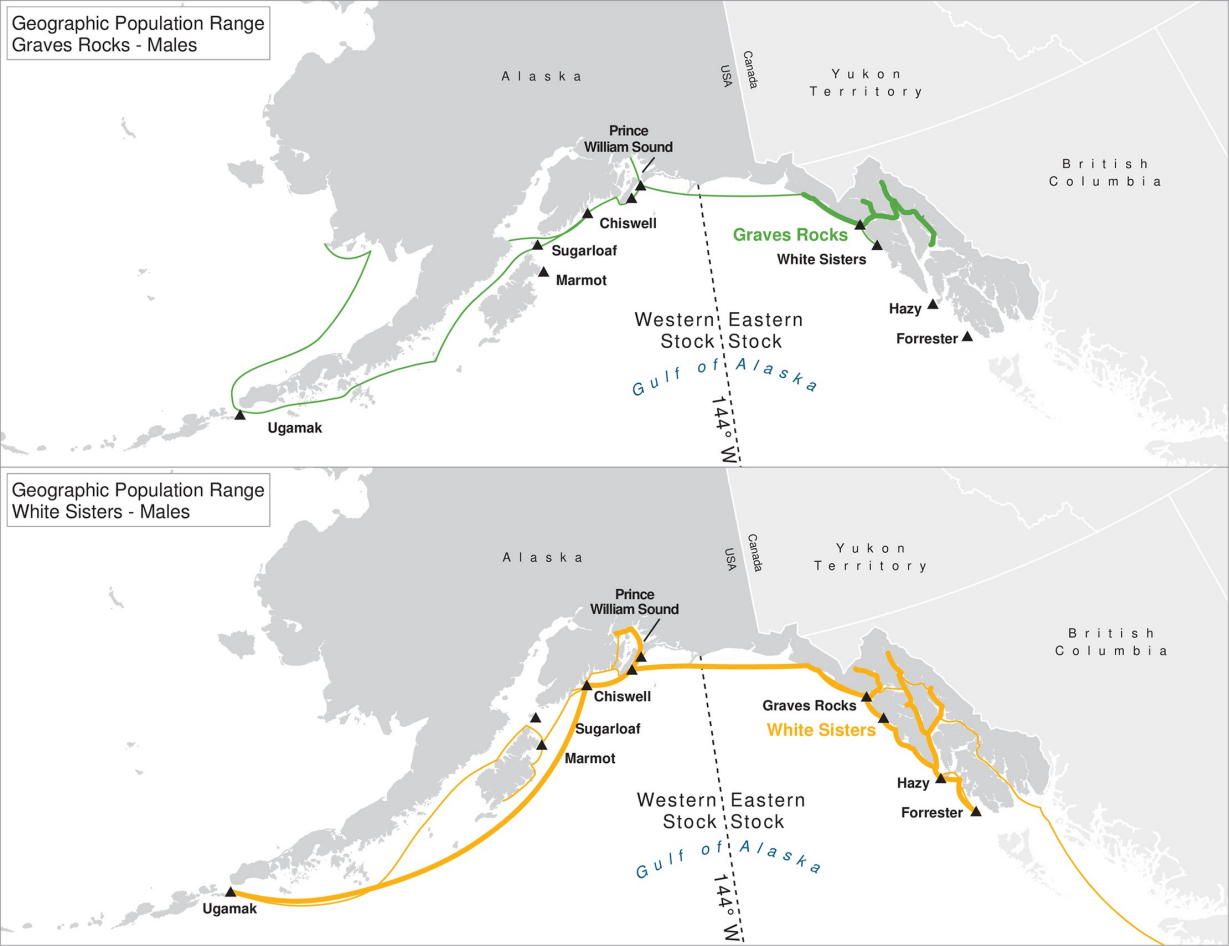
Geographic population range of male Steller sea lions born at Graves Rocks and White Sisters. Population range of male Steller sea lions born at Graves Rocks and White Sisters estimated using minimum spanning tree based on year-round observations of sea lions (of all ages combined) for each natal rookery. Thicker solid line indicates movement between two sites by >1 sea lion; thin lines indicate between-site movement by a single individual.

**Fig 8:**
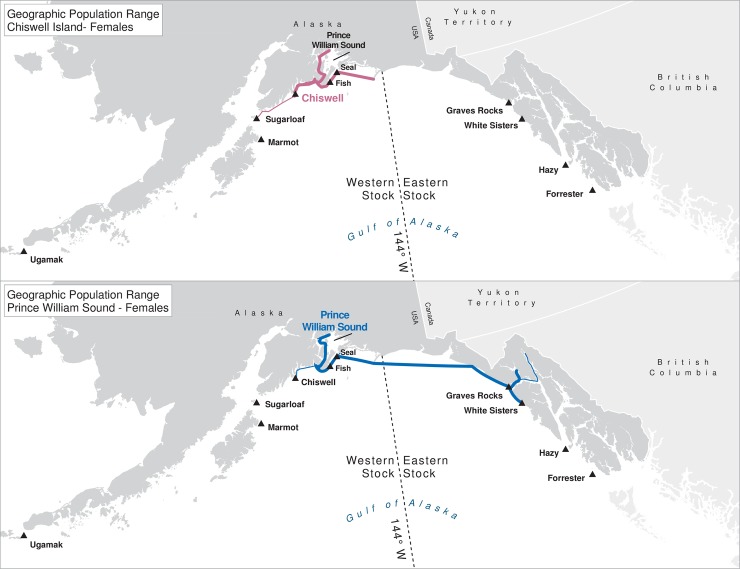
Geographic population range of female Steller sea lions born in Prince William Sound and at Chiswell Island. Population range of female Steller sea lions born in Prince William Sound and at Chiswell Island estimated using minimum spanning tree based on year-round observations of sea lions (of all ages combined) for each natal rookery. Thicker solid line indicates movement between two sites by >1 sea lion; thin lines indicate between-site movement by a single individual.

**Fig 9:**
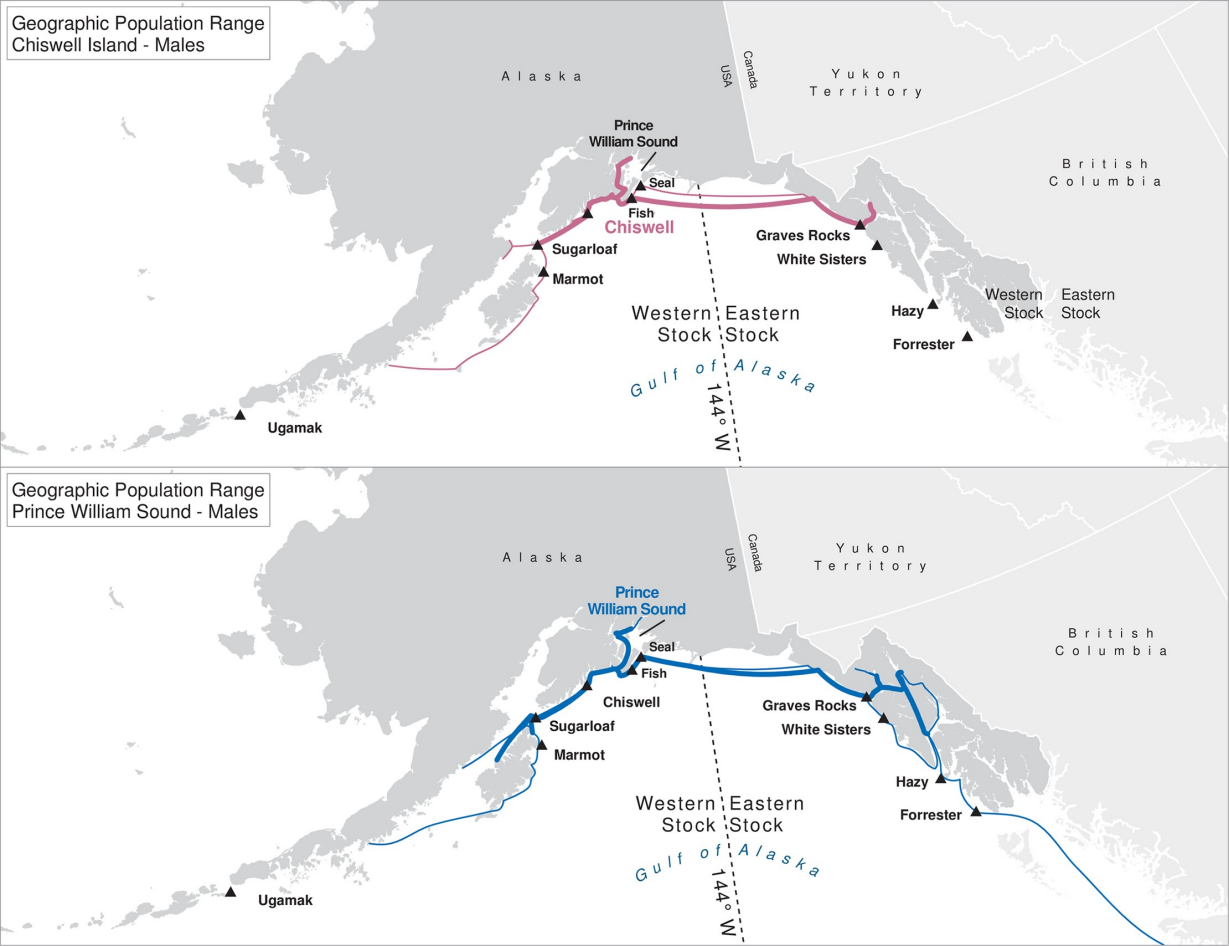
Geographic population range of male Steller sea lions born in Prince William Sound and at Chiswell Island. Population range of male Steller sea lions born in Prince William Sound and at Chiswell Island estimated using minimum spanning tree based on year-round observations of sea lions (of all ages combined) for each natal rookery. Thicker solid line indicates movement between two sites by >1 sea lion; thin lines indicate between-site movement by a single individual.

**Fig 10:**
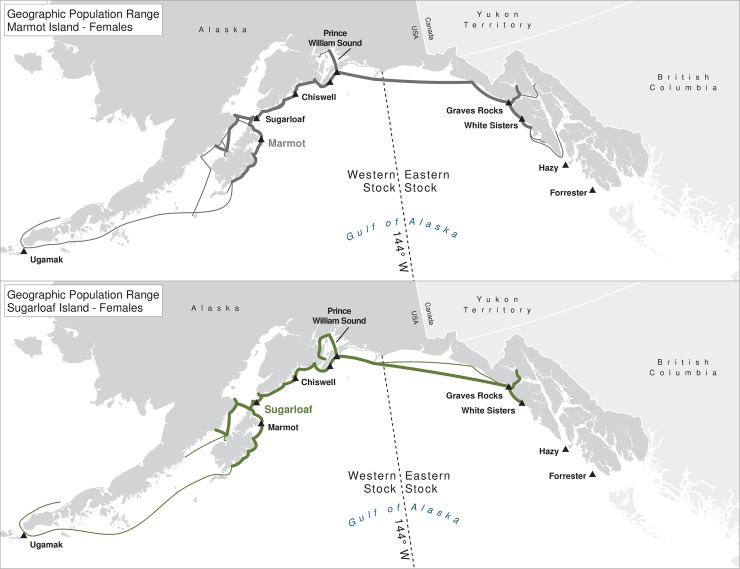
Geographic population range of female Steller sea lions born at Marmot and Sugarloaf Islands. Population range of female Steller sea lions born at Marmot and Sugarloaf Islands estimated using minimum spanning tree based on year-round observations of sea lions (of all ages combined) for each natal rookery. Thicker solid line indicates movement between two sites by >1 sea lion; thin lines indicate between-site movement by a single individual.

**Fig 11:**
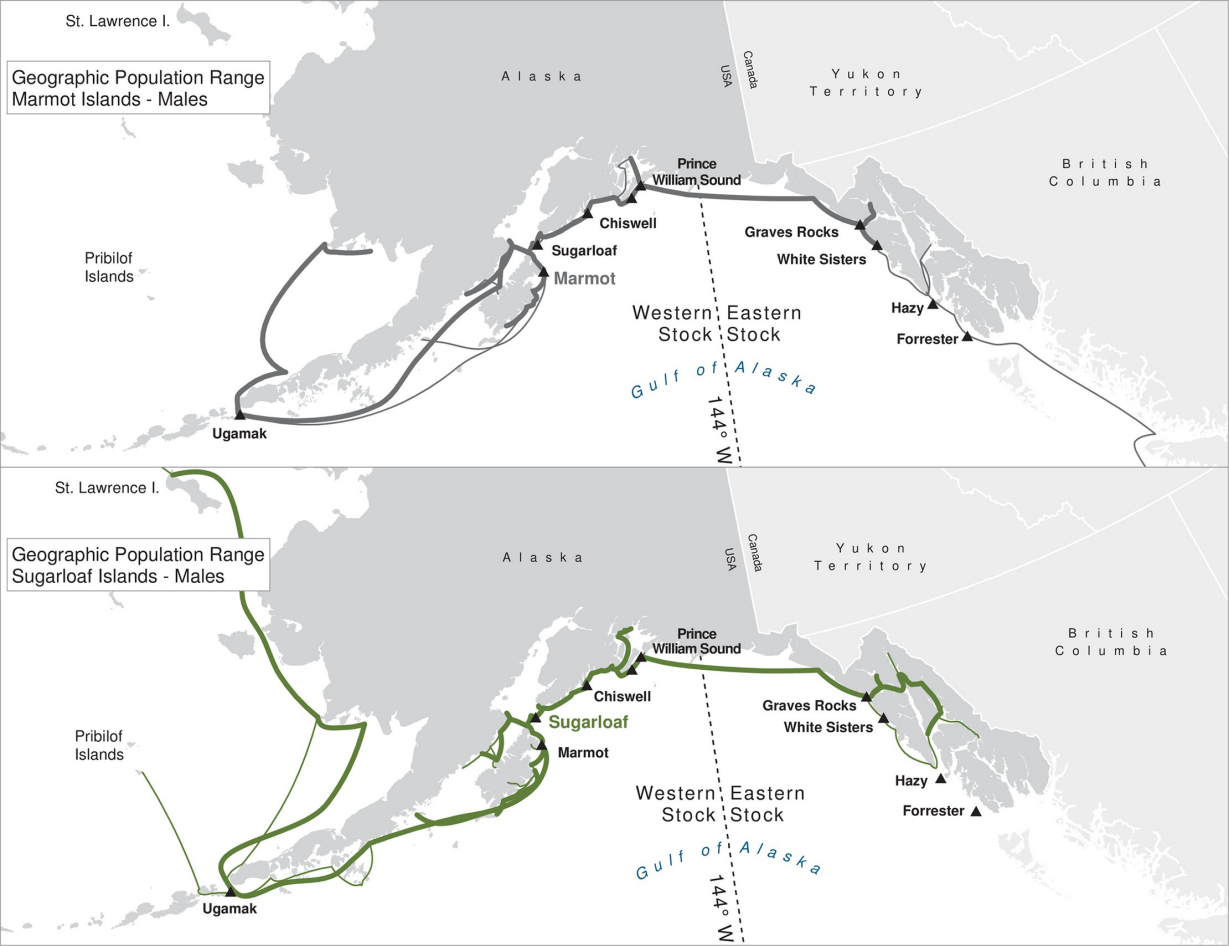
Geographic population range of male Steller sea lions born at Marmot and Sugarloaf Islands. Population range of male Steller sea lions born at Marmot and Sugarloaf Islands estimated using minimum spanning tree based on year-round observations of sea lions (of all ages combined) for each natal rookery. Thicker solid line indicates movement between two sites by >1 sea lion; thin lines indicate between-site movement by a single individual.

**Fig 12:**
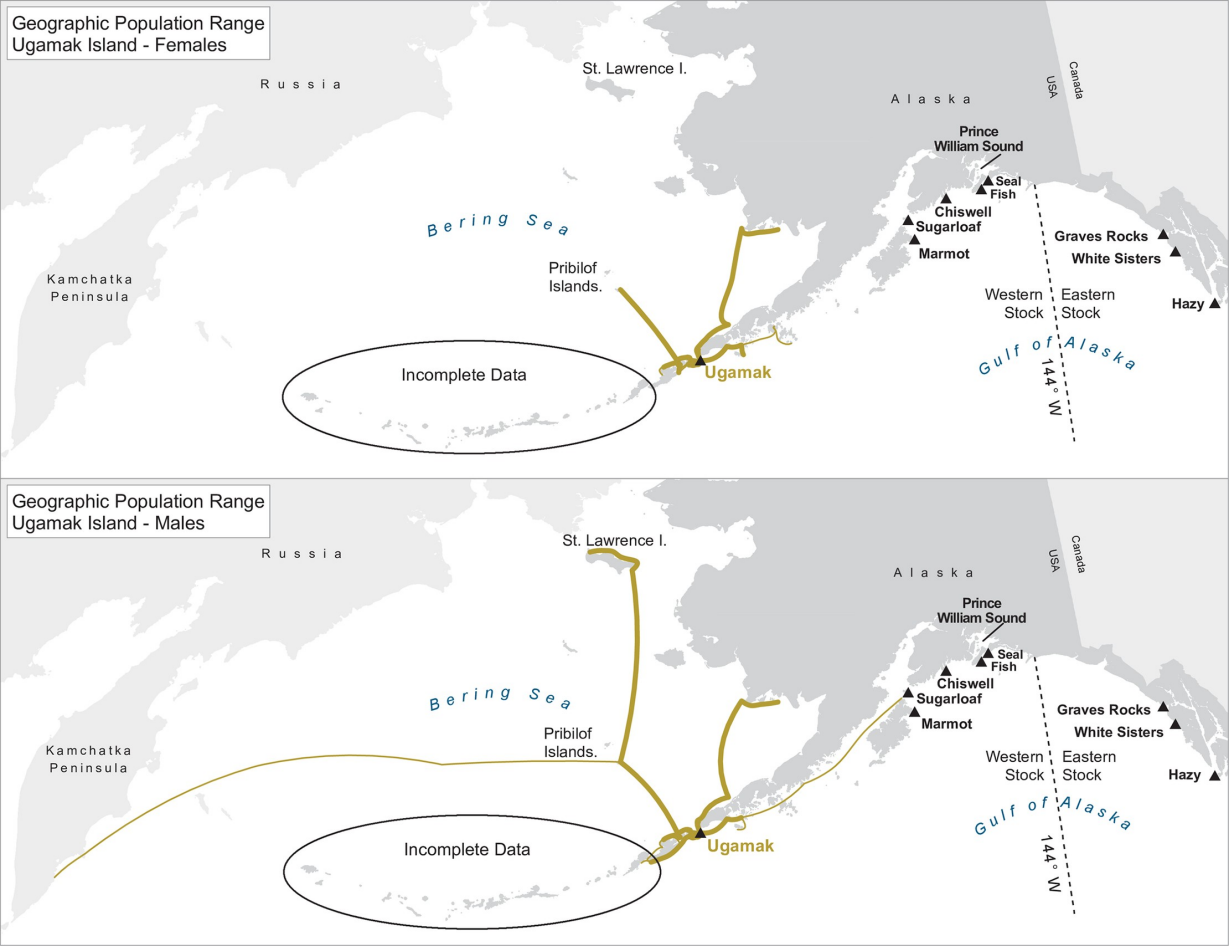
Geographic population range of Steller sea lions born at Ugamak Island. Population range of male and female Steller sea lions born at Ugamak Island estimated using minimum spanning tree based on year-round observations of sea lions (of all ages combined) for each natal rookery. Thicker solid line indicates movement between two sites by >1 sea lion; thin lines indicate between-site movement by a single individual.

For many of the rookeries, geographic ranges of males of all ages combined are much larger than for the three age classes separately, suggesting that males are using different areas at different ages (Table 3). In addition to differing range sizes, the configurations of these ranges differed among rookeries, even those that were near each other (Figs 4–12). As observed in the spatial distribution analysis (objective 1), but here including all observations from both seasons, females from Forrester and Hazy Islands never were observed west of the stock boundary and no males or females from Graves Rocks were seen in southern SEAK. Western females (excluding those born on Ugamak) were either never seen southwest of Kodiak Island (those born in Prince William Sound or on Chiswell Island) or rarely were (those born on Marmot or Sugarloaf Islands); and SSLs from Ugamak Island rarely (n = 5 individuals) traveled more than 200km east of their natal rookery. Western sea lions were seen as far south as Washington State in the Pacific Northwest (Prince William Sound- and Marmot-born males), as far north as Saint Lawrence Island (Sugarloaf-born male), and as far west as Chukotka, Russia (Sugarloaf-born male); Forrester males were observed south to Oregon in the Pacific Northwest, as far north as Saint Lawrence Island, and west to the Pribilof Islands and Seguam Island in the Aleutian Islands (Figs 4–12).

Sites where a single animal from a rookery was seen were often far away from other sites used by SSLs from that rookery. Consequently, including sites where a single marked animal was observed had a large effect on estimated geographic ranges. For females, ranges including sites where a single marked animal was seen were 1.05–5.40 times larger (median 1.51) than ranges with all sites having >1 marked SSL seen (Table 3). Increases were even larger for males (range 1.10–13.42, median 2.02; Table 3). However, the relative patterns in geographic range sizes by natal rookery, sex, and age are similar whether single-observation sites were included or not.

### Objective 4: Geographic population structure

**Fig 13:**
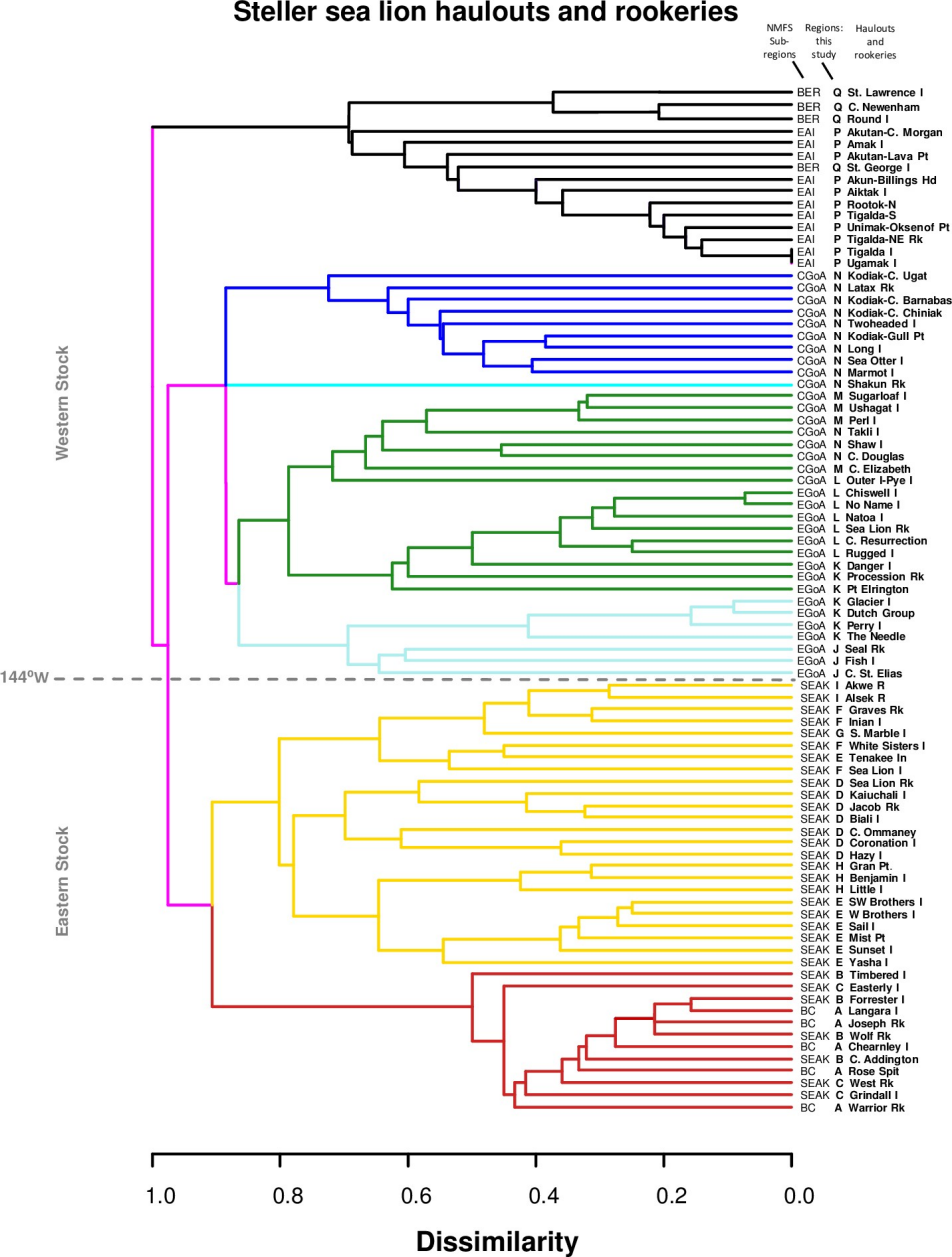
Cluster analysis grouping of sites based on lists of branded sea lions at both sites. All sites included in this figure had ≥10 individual Steller sea lions resighted there. Sites that join at 0 have the exact same individuals seen at both sites (i.e., 0 dissimilarity), while a value of 1 indicates no shared brands (i.e., complete dissimilarity). Colors represent the 7 most distinct groupings with 6 groups and 1 anomalous individual site. Letters before haulout and rookery names indicate the Region containing that site (see Fig 1 for Region designations), and National Marine Fisheries Service (NMFS) Sub-regions shown in abbreviate form before each Region (letter) used in this study.

We restricted the analysis to data for sites where ≥10 individual marked SSLs were seen, because, although results were not appreciably different when all sites were included, sites with very few marked SSLs seen (i.e., n≤3) sometimes grouped with distant sites. As expected, pairs of nearby sites usually had more similar lists of individuals than pairs of distant sites (Fig 13). Seven distinct groupings of sites were observed, including 6 site groups and 1 anomalous individual site, Shakun Rock (color-coded in Fig 13). The expected separation between the two stocks was largely observed, with one exception. Based on genetic data, western SSLs were genetically fairly homogeneous and separate from eastern SSLs [46; 52] (also indicated by the division between yellow/red clusters and blue/green clusters, Fig 13). In contrast to genetic patterns, site use patterns of SSLs using the eastern Aleutians and Bering Sea (western stock, black in Fig 13) were most distinct from other groups, and were even more distinct than all other western groups were from eastern groups. Overall, the site-use clusters corresponded with our choice for regions in objective 1 (Fig 1), with some exceptions. Within the eastern stock, sites in British Columbia and southern SEAK (red) corresponded with Regions A-C (Fig 1), and were distinct from other sites in SEAK (yellow, Fig 13; Regions D-I Fig 1). The red cluster was composed of 83% Forrester Island-branded animals with 13% from Hazy Island. In contrast, the yellow cluster contained large majorities of SSLs from Hazy Islands (73% of those resighted), White Sisters (86%), and Graves Rocks (87%). The substructure within the yellow cluster also largely corresponded to Regions D-I in SEAK in objective 1 (Fig 13). In the west, the cores of regions chosen for objective 1 were similar to groupings based on site use but some regional boundaries shifted. Prince William Sound (light blue; Regions J + and most of K) was distinct from the rest of the western stock, except for three small haulouts (Point Elrington, Danger Island and Procession Rocks) at the southern edge of Region K that grouped with the Kenai Peninsula rather than Prince William Sound (Region L; Fig 13). Sites in the Barren Islands (Region M), which are close to Kodiak Island (Region N), grouped more strongly with sites to the northwest and east along the Kenai Peninsula than the Kodiak Island area (dark green vs. dark blue; Figs 1 and 13). Finally, a few sites north of Kodiak Island across Shelikof Strait (Takli Island, Cape Douglas, Shaw Island) that were originally assigned to Region N, grouped more strongly with Region L, and especially Region M (Fig 13).

### Objective 5: Movement measures and regional population dynamics

Details of our analyses of movement and population measures are found in S2 Text.

**Fig 14:**
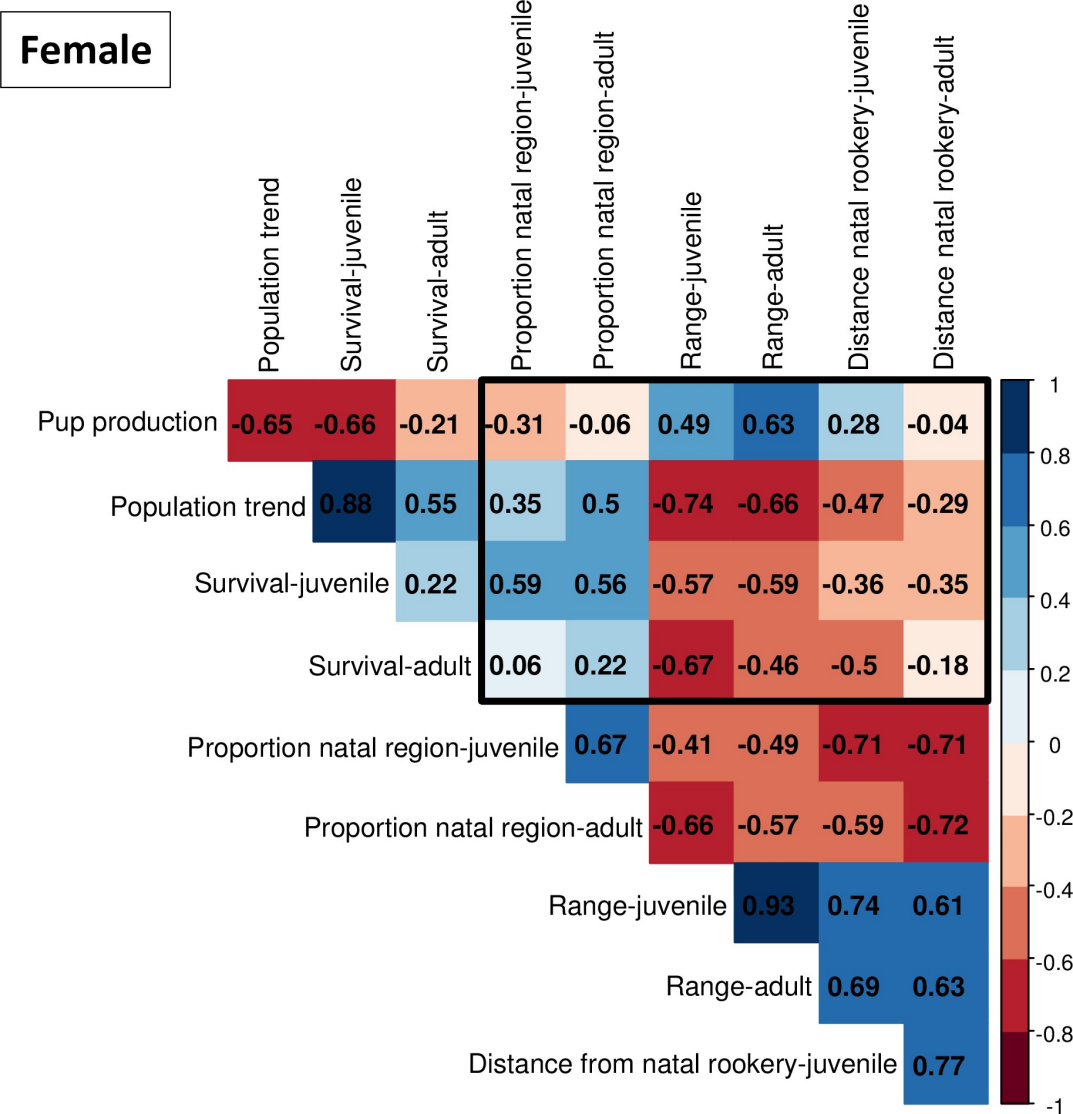
Correlations between female movement measures and population dynamics are shown within the black frame. Movement measures include juvenile and adult proportion in natal region, juvenile and adult geographic range, and juvenile and adult mean distance from natal rookery; population dynamics include pup production, population trend, juvenile and adult survival. Correlations within population dynamics measures are shown outside the frame (top left) and correlations within movement measures are below the frame (bottom right).

**Fig 15:**
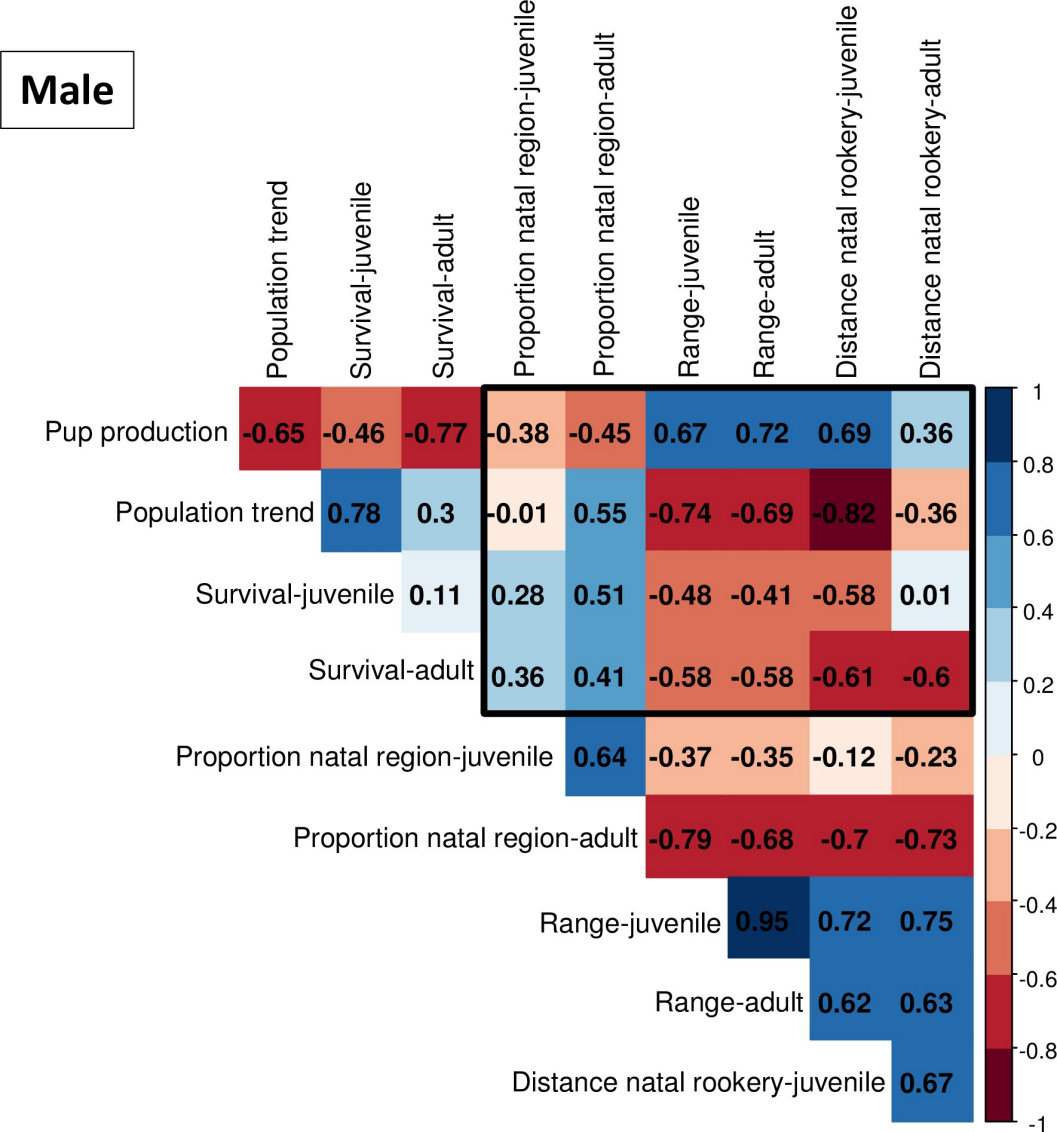
Correlations between male movement measures and population dynamics are shown within the black frame. Movement measures include juvenile and adult proportion in natal region, juvenile and adult geographic range, and juvenile and adult mean distance from natal rookery; population dynamics include pup production, population trend, juvenile and adult survival. Correlations within population dynamics measures are shown outside the frame (top left) and correlations within movement measures are below the frame (bottom right).

We were most interested in correlations between movement and population dynamics measures (within the black frames, Figs 14 and 15), but associations within these 2 sets of statistics are also shown (outside the black frames, Figs 14 and 15). Patterns of correlation were similar for both sexes. Animals from larger rookeries (e.g., larger pup population sizes) had wider dispersion (larger geographic range, greater mean distance from natal site, and lower proportions in the natal region) than animals from smaller rookeries (Figs 14 and 15). Animals from rookeries with stable or slowly increasing populations (i.e., negative or weakly positive pup population trends) and lower survival probabilities had wider dispersion than animals from fast-growing rookeries (higher positive population trends) and rookeries with higher survival (Figs 14 and 15). Pup population trend and survival probabilities were positively correlated, and these two parameters were negatively correlated to pup population size (upper triangles, Figs 14 and 15). As expected, geographic range and mean distance from natal sites were positively correlated, and these two parameters were negatively correlated to proportions in the natal regions (lower triangles, Figs 14 and 15).

## Discussion

Analyses of the summary movement measures (spatial distribution, geographic range, and mean distance from natal rookery) indicate wide variation in rookery-specific movement patterns with SSLs from some rookeries broadly distributed and wide ranging while sea lions from other rookeries remain near or within their natal region; these results were generally consistent across analyses. Rookeries that exhibited similar patterns were not necessarily neighboring rookeries or within the same stock. Instead, we found strong correlations (both positive and negative) among movement patterns and population dynamics measures. For example, SSLs born at Chiswell Island (western stock) and Graves Rocks (eastern stock) had small geographic ranges, the most constricted distributions, and traveled the least distance from their natal rookery. These rookeries also had in common small but increasing populations and high survival rates [38; 60]. Graves Rock is a new rookery (established ~2000 [29; 49] and Chiswell Island was recently re-classified from haulout to rookery in the late 2000s [69]. More localized movements are characteristic of these smaller rookeries where intraspecific competition is lower, although this may also result from higher prey availability (i.e., less movement in especially rich environments). On the other end of the scale, SSLs from larger rookeries where survival rates are lower and population trends were stable or increasing more slowly, were more broadly distributed and farther ranging, suggesting movement patterns could result from density dependence (e.g., competition for food). Forrester Island is the largest rookery in Alaska and is the oldest rookery in SEAK [29]; population growth has slowed at Forrester Island where the non-pup counts have been stable since the late 1970s [29; 42; 49; 70]. Hazy Islands is the next largest rookery ([42] and see S2 Text) with slow growth and low survival rates [29; 60]. SSLs born at these rookeries may be ranging more widely in search of more productive foraging areas or less crowded breeding grounds whereas in the central Gulf of Alaska, populations at Marmot and Sugarloaf Islands were previously very large but are now much reduced from the 1980s and beginning to recover [42]. Limited resources during the period of decline may have resulted in greater dispersal, with animals establishing a pattern of movement away from the natal region and eventually contributing to the establishment of the rookery at Graves Rocks. There is evidence that the eastern stock has been expanding northward for some time, as population growth at Forrester Island stabilized [29; 42; 70]. The most recent genetic data suggest that growth of northern SEAK rookeries (i.e., Graves Rocks and White Sisters) is consistent with positive and negative density dependent emigration of eastern and western animals, respectively [52].

Movement measures for SSLs born in Prince William Sound and at White Sisters generally were intermediate compared to those born at other rookeries. One exception to these patterns was distance measures for Prince William Sound-born females ages 1–5, who were resighted farthest from their natal rookery of all females, yet their geographic range and distribution were intermediate compared to other rookeries. The estimated mean distance Prince William Sound juveniles moved may be positively biased because in the years when Prince William Sound-born SSLs were juveniles, resight effort was low in their natal area but high in more distant areas (such as northern SEAK). We previously found that estimated probabilities of western SSLs moving to the eastern stock were higher for younger Prince William Sound animals compared to other western sea lions [33] and this measure would be less influenced by low resight effort in early years. Prince William Sound females still had a relatively high probability of being in the eastern stock at older ages [33] providing additional support that the patterns we describe here are representative of the true movement patterns.

Age- and sex-specific movement patterns were evident among summary movement measures. During the breeding season, younger SSLs and males were generally more broadly distributed than older animals and females. After accounting for sample size effect, year-round geographic range also varied by age and sex (Table 3): males from all rookeries had larger geographic ranges than their female counterparts, and younger males had larger ranges than older males. These findings of greater dispersion by males and younger animals are consistent with general movement patterns among mammals, especially polygynous species [71; 72], and have been documented in previous studies of SSLs [30; 31; 33]. When unconstrained by territory defense, some male otariids make long distance movements (e.g., Antarctic fur seals, *Arctocephalus gazella*, [73]; Australian fur seals, *Arctocephalus pusillus doriferus*, [74]; northern fur seals, [75]; California sea lions *Zalophus californianus*, [76; 77]; New Zealand sea lions, *Phocarctos hookeri*, [78]; SSLs, [30; 31; 33; 79]) likely in pursuit of prey to recover body reserves after a period of fasting, fighting, and mating. Boyd et al. [73] suggested that male Antarctic fur seals disperse widely, at least in part, to avoid competition with females that may deplete prey resources near the rookery. Not all male SSLs undergo long-distance movements during the non-breeding season (note the relatively small geographic range of males born at Graves Rocks, Table 3) and this is also true of other species. For example, male South American sea lions (*Otaria flavescens*) varied considerably in distances traveled during the non-breeding season: males tagged in the Falkland Islands conducted short foraging trips, acting as central place foragers [80], whereas those marked in Argentina underwent extensive movements to breeding colonies in Patagonia and Uruguay [81]. Juvenile dispersal may be driven by competition for environmental resources or exploration of potential breeding sites.

SSLs that travel long distances overlap with SSLs from within and between stocks, and may be more vulnerable to environmental risks compared to animals that remain near or within their natal region. Inherent risks associated with long distance movements include, for example, greater exposure to predation, pollutants, and disease [78; 82–85]. Animals foraging in the western Aleutian Islands may be at risk of increased exposure to mercury [82]. SSL pups in this region were found to have elevated levels of total mercury concentrations, and the highest levels compared with pups sampled throughout Alaska, a reflection of the prey available in the region that was consumed by their mothers [82]. The eastern Gulf of Alaska has been identified as a region of high mortality for juvenile SSLs due to predation [85] and so SSLs traveling through this area may be at greater risk. New Zealand sea lions have a small, threatened population with a limited breeding range where males disperse both during and after the breeding season [78; 83]. Dispersing males may have been vectors in several disease outbreaks that resulted in high pup mortality in New Zealand sea lions [78; 84]. Far-ranging movements that increase an animal’s exposure to risk factors outside the natal region may have contributed to the lower survival rates and slower growth we found among rookeries where animals are more dispersed. In SEAK, survival was reduced among males that dispersed long distances [60].

The importance of northern SEAK to western SSLs, especially Regions F, G, and I (and Regions E and H if males only are considered, Fig 1) where sea lions from both stocks mix and/or breed, is evident from the summary movement measures and previous work [33], including genetic studies [52]. Region I (Fig 1) contains a single small haulout (<300 SSLs, [49]) during the breeding season that is infrequently surveyed (every ~5 years), resulting in low resight densities. However, this region is important to sea lions in late winter and spring, drawn to the region by eulachon (*Thaleichthys pacificus*) which spawn in local rivers [86]. During March-April, there is a large but transitory influx of sea lions (>4,500 in some years) with animals hauling out in 5+ locations; male and female western stock SSLs account for approximately ~20% of the branded animals using this region (ADF&G and U.S. Forest Service unpublished data). Region F supports the greatest overlap between the two stocks, having the highest diversity index; adjacent regions (E: Frederick Sound-Chatham Strait, G-H: Glacier Bay-Lynn Canal) were among the next most diverse. Western females generally were dispersed eastward from their natal rookery, both within their natal stock and across the stock boundary and were regularly seen in northern SEAK during the breeding season. The tendency toward eastward movement by females born at most western rookeries suggests a payoff for unidirectional travels. Whether this pattern was established during the population decline or in the period of recovery is unclear, but the pattern continues to present time. Northern SEAK may be especially important to Prince William Sound-born females: they never travelled to Sugarloaf or Marmot Islands (~350km to the southwest), and almost never traveled to nearby Chiswell Island (~150km to the southwest) but regularly traveled to northern SEAK where they continue to give birth at Graves Rocks (>600km to the southeast; [33], ADF&G unpublished data). The long-distance movements by Prince William Sound females to eastern rookeries rather than to rookeries nearby differs from observations of eastern females, which, if they give birth at a non-natal site, usually chose the nearest rookery to their natal rookery [37]. The movement pattern of Prince William Sound females seemingly is not a function of rookery size, age, or growth patterns at alternative sites. Of the closest rookeries, Sugarloaf and Marmot Islands are older and larger, but with much reduced, though currently increasing, populations with large ranges, suggesting past or current resource limitations. Chiswell Island is a small, growing, recently established rookery with a small range and high survival, suggesting high resource availability. So, likely availability of resources nearby is not the only factor driving the movements. Like Chiswell Island, the Graves Rocks rookery (in Region F), which is the destination for many Prince William Sound females, is a relatively recently founded rookery, has high population growth and survival, and a small geographic range, again suggesting high resource availability. It is possible that use of northern SEAK, including Region F, by Prince William Sound females is cultural. Juvenile SSLs typically stay with their mothers for 1–3 years [38; 39] and might learn movement patterns from them. If past generations of Prince William Sound females moved to northern SEAK (as suggested by genetic evidence [52]) in response to resource scarcity in the west, such patterns could persist even after resources in the west improve. Sugarloaf and Marmot Islands would not have been good alternatives to Prince William Sound rookeries as they also had major population declines, and Chiswell Island had not yet formed as a rookery, also making it unavailable as an alternate choice for reproduction, leaving northern SEAK as the nearest high resource area available to emigrants from the west. Mathews et al. [49] suggested that improving prey resources within Glacier Bay (Region G in northern SEAK), due to new habitat availability after deglaciation, and improved salmon fisheries management practices in the 1970s in the Icy Strait—Cross Sound region (Regions F and G) may be factors in increased numbers of SSLs within this area. Satellite-tagged pup and juvenile SSLs captured within Glacier Bay remained primarily within Regions F and G, whereas pup and juvenile SSLs captured elsewhere in SEAK were farther ranging [87]. Collectively these factors suggest Region F has abundant resources and high quality habitat that western sea lions seek out, even if distant from their natal region.

Northern SEAK is important to eastern sea lions. SSLs born locally tend to remain in the region: animals born at White Sisters and Graves Rocks rarely or never traveled to southern SEAK rookeries, and almost never traveled west of the stock boundary. Although sample size was small, natal philopatry of females born at Graves Rocks was 100% and these females only ever pupped at Graves or White Sisters rookeries [37]. Similarly, 90% of females from White Sisters that were ever observed with pup, first pupped at Graves or White Sisters [37]. Males born at Graves Rocks were documented as breeding bulls only at their natal rookery, and White Sisters males only at Graves Rocks or White Sisters rookeries, whereas males from Forrester and Hazy Islands held territories at several or all rookeries in SEAK [41]. Survival of southern-born SSLs (from Forrester and Hazy Islands) was higher for those animals that used northern SEAK than those that did not use the area [60].

Genetic and morphological data demonstrate that SSLs in the eastern and western stocks are long-separated populations [46; 88; 89] that have recently (since 1990, [29]) established a sympatric mixing zone within the eastern stock, in northern SEAK [33; 52]. Such patterns of long-separated populations re-establishing sympatry and mixing zones have also been demonstrated in a number of terrestrial species (e.g., black bears, *Ursus americanus*, [90; 91]; bobcats, *Lynx rufus*, [92]; martens, *Martes americana*/*Martes caurina*, [93]). Asymmetric movement and colonization from one population towards another, narrowing the separation between the two populations, has been reported elsewhere for SSLs with the western stock establishing a rookery on the Commander Islands (the western-most islands in the Aleutian Islands chain), narrowing the gap with the Asian stock [27; 88]. However, genetic patterns that strongly support population separation might change too slowly to reflect current interactions. Based on genetics, the western stock is defined as a single population from the eastern Gulf of Alaska west through all of Russia, with an additional split proposed between the Kamchatka Peninsula and the Commander Islands [46; 88]. Based on our movement data, however, SSLs born in some regions of the western stock have very little interaction with each other yet have strong patterns of spatial-temporal overlap with animals born in the eastern stock. The geographic range of females born at Ugamak Island primarily includes sites to the north and west of their natal region with no individuals from Ugamak of either sex observed east of Kodiak Island and only one Ugamak-born animal, a male, resighted within Region N. Yet, other western females generally move within or to the east of their natal region, extending into the eastern stock and reproducing there (all but Chiswell females), but generally not overlapping the range used by Ugamak SSLs. By contrast, eastern females moved east and west of their natal region within the eastern stock, but to date, only two eastern females in this study have been documented in the western stock, and none have been observed giving birth there. Population structure based on cluster analysis supported these findings with eastern Aleutian Island animals being the most distinct group, more dissimilar than other western animals are compared to eastern animals. Note however that unlike our data from the eastern stock where we have marked samples from 4 of the 5 rookeries in SEAK, in the western stock we have samples from a far smaller proportion of rookeries. Ugamak Island is 966 km west of Marmot Island and is the closest rookery to the west where pups have been marked. However, there are 12 rookeries between Marmot and Ugamak where no pups have been marked [94]. Although the distribution and range of Ugamak-marked SSLs is very distinct from rookeries in the central and eastern Gulf of Alaska, the distribution of animals at intervening rookeries might overlap with both Ugamak animals and those from farther east, making the difference appear less abrupt. SSLs born at Prince William Sound rookeries were also fairly distinct from other western animals.

Knowledge of mesoscale spatial distributions and geographic ranges of SSLs from different natal rookeries suggests finer spatial structure than the current east-west stock designation, and is useful in several management contexts. Our data based on resightings of individually-marked SSLs show somewhat different structure than our chosen regions for objective 1 (Fig 1), as well as the regions and Rookery Cluster Areas of Fritz et al. [42], suggesting that regional boundaries for future analyses could be modified to better reflect population structure and processes. Because SSLs within the refined regions are more likely to be interacting and to be subject to similar environmental conditions, greater precision of population trend estimates may be possible using the refined regional designations. Inter-regional movement also has the potential to affect population trend estimates with one region essentially subsidizing another. For example, Fritz et al. [42] suggested that net movement of ~1200 animals from the central to the eastern Gulf of Alaska could have affected estimates of regional non-pup population trends. If NMFS were to update the recovery plan [95] (now 10 years old) for the western stock, our results would provide valuable information for redefining recovery and population trend analysis regions that better reflect dispersal and population structure. For instance, the border between the eastern and central Gulf of Alaska could be shifted to the southwest to include Sugarloaf Island and sites along the northeastern edge of the Alaska Peninsula. However, regional structure west of Marmot Island cannot be currently addressed due to lack of marking and resighting studies between Marmot and Ugamak Islands [42].

Beginning in the early 1990s, the NMFS has enacted a series of management measures designed to promote the recovery of SSLs, including the designation of critical habitat under the Endangered Species Act in 1993 [95]. Throughout most of the range of the western stock in Alaska, NMFS has also taken steps designed to reduce competition between federally managed groundfish fisheries and SSLs, including exclusion of certain fisheries near rookeries and some major haulouts, and temporal/spatial allocation of some fishing quotas to minimize likelihood of localized depletions of prey [95–98]. None of the fishery restrictions apply to haulouts and rookeries within the eastern stock, although northern SEAK is important to breeding and foraging western SSLs. The division between the eastern and western stocks was established in 1997 [48], based on population trends, and genetic information that did not include samples from Graves Rocks (which at that time was not yet a rookery), but did include samples from White Sisters which was just emerging as a rookery [29; 46; 47; 49]. Recent genetic data from pups born at Graves Rocks and White Sisters indicated that a higher proportion of pups born at Graves Rocks had western origins, whereas a higher proportion of pups born at White Sisters had eastern origins [52]. The location of these rookeries within the eastern stock, but with colonization and reproduction by SSLs from both stocks, brings into question the current stock boundary and existing management units. Whether this shared region of apparent high quality habitat and abundant resources warrants protections in relation to fisheries or other activities is worth consideration. Our results provide resource managers with areas on the northern SEAK outer coast and Glacier Bay (Regions F, G, and I, Fig 1), used regularly by western animals, which could be considered for additional protection to promote recovery of the western stock. This greater protection could be extended to Lynn Canal and Frederick Sound (Regions E and H, Fig 1) to benefit western males. Currently the stock boundary is somewhat arbitrary, given our current knowledge of SSL movements and genetics. It may be time to redefine the stock boundary and reassess associated management strategies.

## Supporting information

S1 Text Effect of sample size on spanning tree estimates of geographic range. (DOCX)

S2 Text Estimating population trend: Methods and results. (DOCX)

S1 Fig Spatial distribution of juvenile female Steller sea lions born in the eastern stock during the breeding season. Breeding season distribution of juvenile female Steller sea lions born in the eastern stock based on an index of resight density. Resight density is the proportion of Steller sea lions seen within a region relative to the total number of sea lions from that natal rookery seen anywhere. Regions J through Q are not included on eastern stock female maps; refer to Table 2 for densities of these areas.
(TIF)

S2 Fig Spatial distribution of adult female Steller sea lions born in the eastern stock during the breeding season. Breeding season distribution of adult female Steller sea lions born in the eastern stock based on an index of resight density. Resight density is the proportion of Steller sea lions seen within a region relative to the total number of sea lions from that natal rookery seen anywhere. Regions J through Q are not included on eastern stock female maps; refer to Table 2 for densities of these areas.
(TIF)

S3 Fig Spatial distribution of juvenile male Steller sea lions born in the eastern stock during the breeding season. Breeding season distribution of juvenile male Steller sea lions born in the eastern stock based on an index of resight density. Resight density is the proportion of Steller sea lions seen within a region relative to the total number of sea lions from that natal rookery seen anywhere. Regions O, P, and Q are not included on eastern stock male maps; refer to Table 2 for densities of these areas.
(TIF)

S4 Fig Spatial distribution of sub-adult male Steller sea lions born in the eastern stock during the breeding season. Breeding season distribution of sub-adult male Steller sea lions born in the eastern stock based on an index of resight density. Resight density is the proportion of Steller sea lions seen within a region relative to the total number of sea lions from that natal rookery seen anywhere. Regions O, P, and Q are not included on eastern stock male maps; refer to Table 2 for densities of these areas.
(TIF)

S5 Fig Spatial distribution of adult male Steller sea lions born in the eastern stock during the breeding season. Breeding season distribution of adult male Steller sea lions born in the eastern stock based on an index of resight density. Resight density is the proportion of Steller sea lions seen within a region relative to the total number of sea lions from that natal rookery seen anywhere. Regions O, P, and Q are not included on eastern stock male maps; refer to Table 2 for densities of these areas.
(TIF)

S6 Fig Spatial distribution of juvenile female Steller sea lions born in the western stock during the breeding season. Breeding season distribution of juvenile female Steller sea lions born in the western stock based on an index of resight density. Resight density is the proportion of Steller sea lions seen within a region relative to the total number of sea lions from that natal rookery seen anywhere. Regions A through C and O through Q are not included on the western stock female maps; refer to Table 2 for densities.
(TIF)

S7 Fig Spatial distribution of adult female Steller sea lions born in the western stock during the breeding season. Breeding season distribution of adult female Steller sea lions born in the western stock based on an index of resight density. Resight density is the proportion of Steller sea lions seen within a region relative to the total number of sea lions from that natal rookery seen anywhere. Regions A through C and O through Q are not included on the western stock female maps; refer to Table 2 for densities.
(TIF)

S8 Fig Spatial distribution of juvenile male Steller sea lions born in the western stock during the breeding season. Breeding season distribution of juvenile male Steller sea lions born in the western stock based on an index of resight density. Resight density is the proportion of Steller sea lions seen within a region relative to the total number of sea lions from that natal rookery seen anywhere. Regions A and P are not included on western stock male maps; refer to Table 2 for densities.
(TIF)

S9 Fig Spatial distribution of sub-adult male Steller sea lions born in the western stock during the breeding season. Breeding season distribution of sub-adult male Steller sea lions born in the western stock based on an index of resight density. Resight density is the proportion of Steller sea lions seen within a region relative to the total number of sea lions from that natal rookery seen anywhere. Regions A and P are not included on western stock male maps; refer to Table 2 for densities.
(TIF)

S10 Fig Spatial distribution of adult male Steller sea lions born in the western stock during the breeding season. Breeding season distribution of adult male Steller sea lions born in the western stock based on an index of resight density. Resight density is the proportion of Steller sea lions seen within a region relative to the total number of sea lions from that natal rookery seen anywhere. Regions A and P are not included on western stock male maps; refer to Table 2 for densities. On map of Chiswell males age 9+, note that this represents only 3 individuals (all other maps represent ≥10 individuals).
(TIF)

S1 Table Mean and maximum distance between Steller sea lion locations and natal rookery during breeding season. Values are mean distance (95% CI), maximum distance, and sample size.
(DOCX)
