# Supplementary material for: Spatial distribution, movements, and geographic range of Steller sea lions (Eumetopias jubatus) in Alaska
Source: PLoS One. 2018 Dec 26;13(12):e0208093. doi: 10.1371/journal.pone.0208093 (PMC6306159; doi:10.1371/journal.pone.0208093)
Supplement: S1 Text — (DOCX) [file pone.0208093.s001.docx]

S1 Text: Effect of sample size on spanning tree estimates of geographic range.

To examine effect of sample size on range estimates, we selected random samples of SSLs (separately by sex) from either Forrester or Sugarloaf (both of which have large sample sizes). We then used resights of these animals to compile lists of used sites and calculated the geographic range for each random sample. The random samples ranged in size from 25 – 150 in increments of 25 (to approximate sample sizes from other rookeries). We repeated this process 100 times for each combination of rookery (*i.e.*, Forrester or Sugarloaf), sex, and sample size to estimate variation in range size for a fixed sample size. If sample size affected range estimates for Forrester-Sugarloaf animals, we adjusted range size to a "relative" size for all other sex-rookery combinations (other than those from Forrester and Sugarloaf) by dividing the observed range size for each group by the pooled Forrester-Sugarloaf range size for a comparable sample size. We calculated these ratios for each of the 200 replicates (100 each from Forrester and Sugarloaf), then calculated the mean ratio (and 95% confidence intervals) as a sample-size adjusted measure of relative range size for each rookery.

Based on the random subset analyses, geographic range estimates increased with increasing sample size of individuals included in the analysis (Fig A, below). After adjusting for sample size, the relative geographic ranges (compared to Forrester-Sugarloaf) for females based on natal rookery were 0.33 for Chiswell, 0.55 for Graves, and about 0.67 for Prince William Sound and White Sisters (Table 3). Relative range sizes were only slightly smaller or not smaller (confidence intervals overlapped 1) for females born at Ugamak, Hazy, and Marmot Islands (Table 3). The relative ranges for males based on natal rookery were 0.34 for Chiswell, 0.41-0.48 for Graves and Prince William Sound, and 0.62-0.66 for Ugamak and Hazy, and only slightly smaller (or not smaller) for White Sisters and Marmot (Table 3). Note that Ugamak geographic ranges were likely underestimated due to incomplete sampling west of Ugamak Island.

**Fig A. Estimated geographic ranges based on reduced size samples from Forrester and Sugarloaf Islands.**

Results from females are in gray and males in black. For each sample size, narrow lines extend from the minimum to the maximum, wide lines are the interquartile range, and gap is the median. Boxes to the right are the observed geographic ranges for females (n=247) and males (n=264) from Forrester Island.
