# Supplementary material for: Spatial distribution, movements, and geographic range of Steller sea lions (Eumetopias jubatus) in Alaska
Source: PLoS One. 2018 Dec 26;13(12):e0208093. doi: 10.1371/journal.pone.0208093 (PMC6306159; doi:10.1371/journal.pone.0208093)
Supplement: S2 Text — (DOCX) [file pone.0208093.s002.docx]

S2 Text. Estimating population trend: methods and results.

We estimated population trend based on pup counts collected by the Marine Mammal Lab from 2000 to 2015 for each of the 10 rookeries using a negative binomial generalized linear model (glm.nb in R package MASS; [[1](#_ENREF_1); [2](#_ENREF_2)]); negative binomial models were used to accommodate over dispersion in the data. Rookeries had 5–9 pup counts available for the 2000-2015 interval. Steller sea lion (SSL) birth dates were later in the west than east [[3](#_ENREF_3)] and so date effect may differ between stocks. Consequently, we fit 4 models related to a potential date effect: 1) no date effect, 2) linear date effect (same effect for east and west), 3) linear and quadratic date effects (same effects for east and west), and 4) separate linear and quadratic effects for east and west. A year effect to estimate trend was in all 4 models. Population trend estimates were taken from the best model as judged by the model with the lowest AIC value. Model validation was conducted as described by Zuur et al. [[4](#_ENREF_4)]. Pup population size was the sum of pups produced per rookery from 2000–2015 based on the predicted pup counts from the population trend model. Population trend and pup population size for Fish Island and Seal Rocks were averaged (weighted by pup population size per rookery) and summed, respectively, to produce estimates for the pooled region, Prince William Sound. We used published rookery- and sex- specific estimates for juvenile (cumulative survival from age 0 to 4) and adult (annual survival at age 7 for females and 9 for males) survival probabilities during these same years [[[5-7](#_ENREF_5)] see S1 Table in Maniscalco et al. [[7](#_ENREF_7)], averaging values from 2003–13]. Movement measures for each rookery were calculated as previously described with the distances to natal rookeries at age 2 used for juveniles, age 7 used for adult females, and age 9 used for adult males.

The negative binomial generalized linear model fit the data well as determined by model diagnostics. The best of 4 models (Table S2) included a date effect on pup counts that was linear and differed by stock: increasing slightly with date in the west and decreasing with date in the east. However, removal of stock-specific date effect had little effect on population trend estimates. Estimates of annual pup count trends (s.e.) from 2000-2015 were -0.004 (se=0.010), 0.026 (se=0.010), 0.051 (se=0.011), 0.108 (se=0.012) in SEAK (respectively: Forrester Islands, Hazy Islands, White Sisters, Graves Rock), and were 0.040 (se=0.008), 0.043 (se=0.013), 0.048 (se=0.008), 0.020 (se=0.011), and 0.031 (se=0.009) in the west (respectively: Prince William Sound, Chiswell, Sugarloaf, Marmot, and Ugamak Islands). Pup population sizes relative to Forrester Islands were 0.44, 0.18 and 0.08 for other SEAK rookeries (respectively: Hazy Islands, White Sisters, Graves Rock) and were 0.23, 0.02, 0.18, 0.14 and 0.22 for western rookeries (respectively: Prince William Sound, Chiswell, Sugarloaf, Marmot, and Ugamak Islands).

Table S2. Model selection results for trend estimation models. Stock = east versus west

| Predictor variables | AIC | Model wt. |
| --- | --- | --- |
| Rookery*yr + date*stock + date^2^*stock | 791.904 | 0.275 |
| Rookery*yr + date*stock | 789.993 | 0.715 |
| Rookery*yr + date | 799.901 | 0.005 |
| Rookery*yr | 799.999 | 0.005 |

References

1.R Development Core Team. R: A language and environment for statistical computing. Vienna, Austria: R Foundation for Statistical Computing; 2017.

2.Fritz LW, Sweeney K, Lynn M, Gelatt T, Gilpatrick J, Towell R. Counts of Alaska Steller sea lion pups conducted on rookeries in Alaska from 1961-06-22 to 2015-07-18 (NCEI Accession 0128189). Version 2.4. NOAA National Centers for Environmental Information. Dataset. doi:10.7289/V5862DDR. 2015.

3.Pitcher KW, Burkanov VN, Calkins DG, Le Boeuf BJ, Mamaev EG, Merrick RL, et al. Spatial and Temporal Variation in the Timing of Births of Steller Sea Lions. J Mammal. 2001;82(4):1047-53. doi: 10.1644/1545-1542(2001)082<1047:satvit>2.0.co;2.

4.Zuur AF. Mixed effects models and extensions in ecology with R. New York: Springer; 2009.

5.Hastings KK, Jemison LA, Gelatt TS, Laake JL, Pendleton GW, King JC, et al. Cohort effects and spatial variation in age‐specific survival of Steller sea lions from southeastern Alaska. Ecosphere. 2011;2(10):1-21.

6.Fritz LW, Towell RG, Gelatt TS, Johnson DS, Loughlin TR. Recent increases in survival of western Steller sea lions in Alaska and implications for recovery. Endanger Species Res. 2014;26(1):13-24.

7.Maniscalco JM, Springer AM, Adkinson MD, Parker P. Population trend and elasticities of vital rates for Steller sea lions (*Eumetopias jubatus*) in the Eastern Gulf of Alaska: a new life-history table analysis. PLoS ONE. 2015;10. doi: 10.1371/journal.pone.0140982.
