# Supplementary figures and images for: Spatial distribution, movements, and geographic range of Steller sea lions (Eumetopias jubatus) in Alaska
Source: PLoS One. 2018 Dec 26;13(12):e0208093. doi: 10.1371/journal.pone.0208093 (PMC6306159; doi:10.1371/journal.pone.0208093)

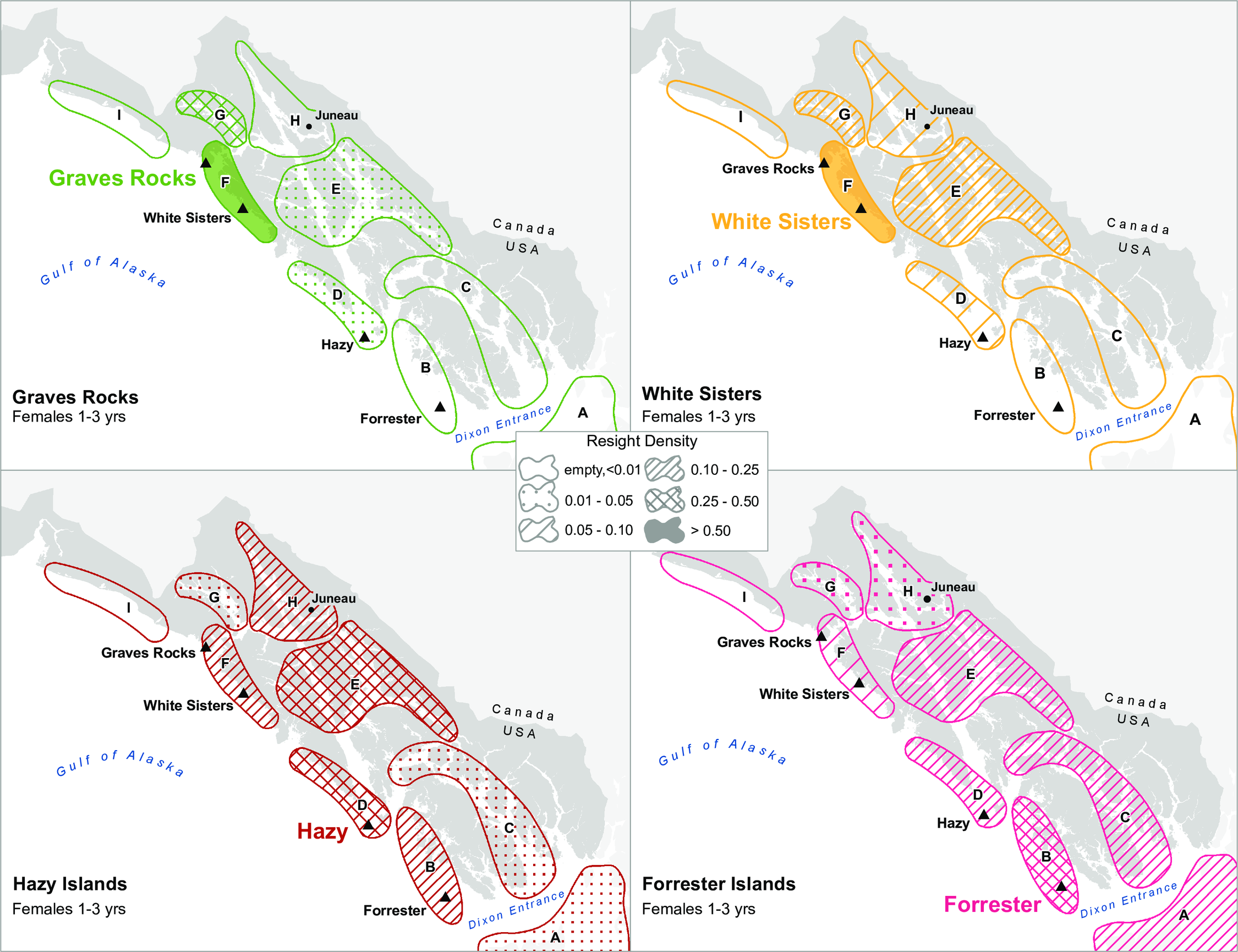

Supplement: S1 Fig — Breeding season distribution of juvenile female Steller sea lions born in the eastern stock based on an index of resight density. Resight density is the proportion of Steller sea lions seen within a region relative to the total number of sea lions from that natal rookery seen anywhere. Regions J through Q are not included on eastern stock female maps; refer to Table 2 for densities of these areas. (TIF) [file pone.0208093.s003.tif]

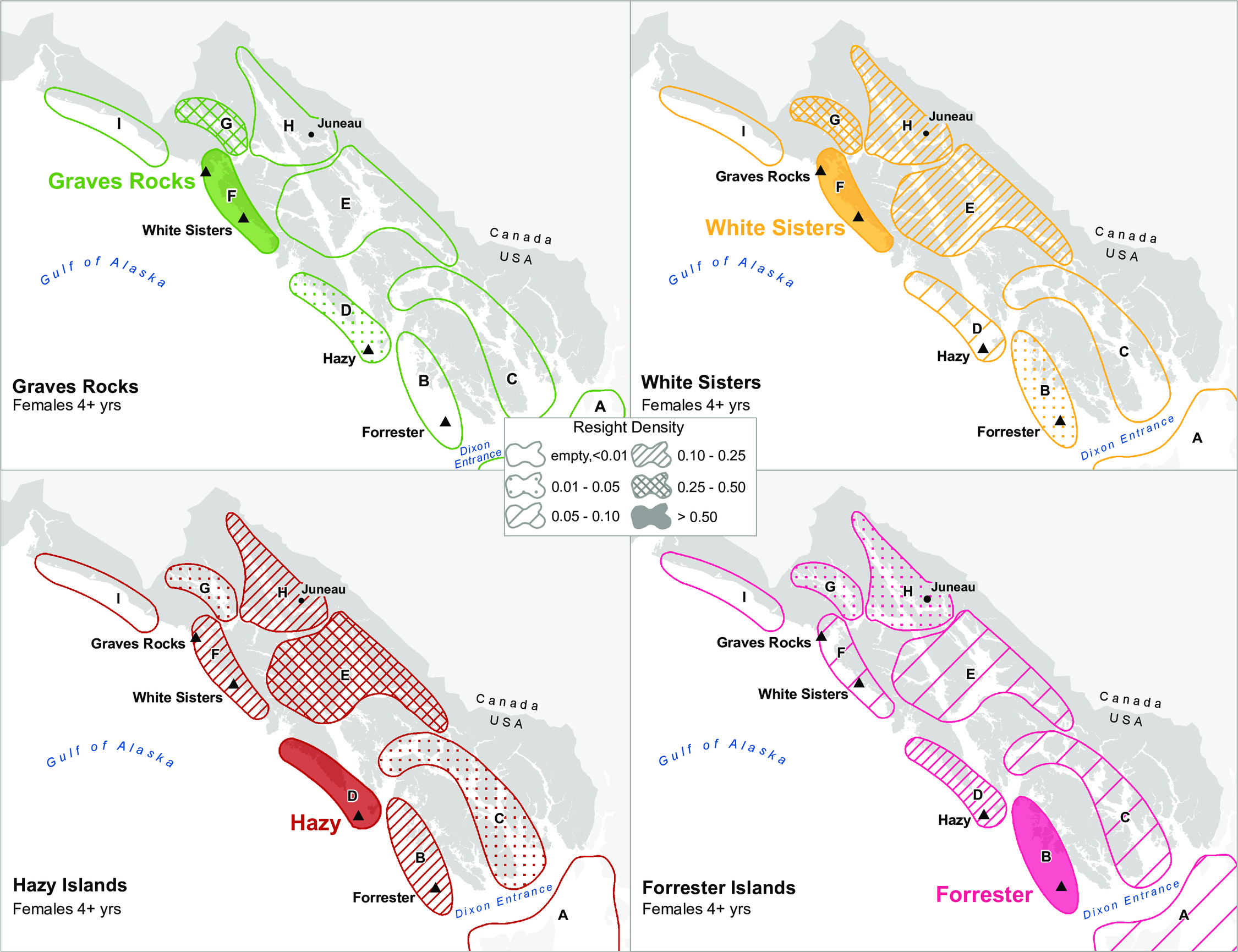

Supplement: S2 Fig — Breeding season distribution of adult female Steller sea lions born in the eastern stock based on an index of resight density. Resight density is the proportion of Steller sea lions seen within a region relative to the total number of sea lions from that natal rookery seen anywhere. Regions J through Q are not included on eastern stock female maps; refer to Table 2 for densities of these areas. (TIF) [file pone.0208093.s004.tif]

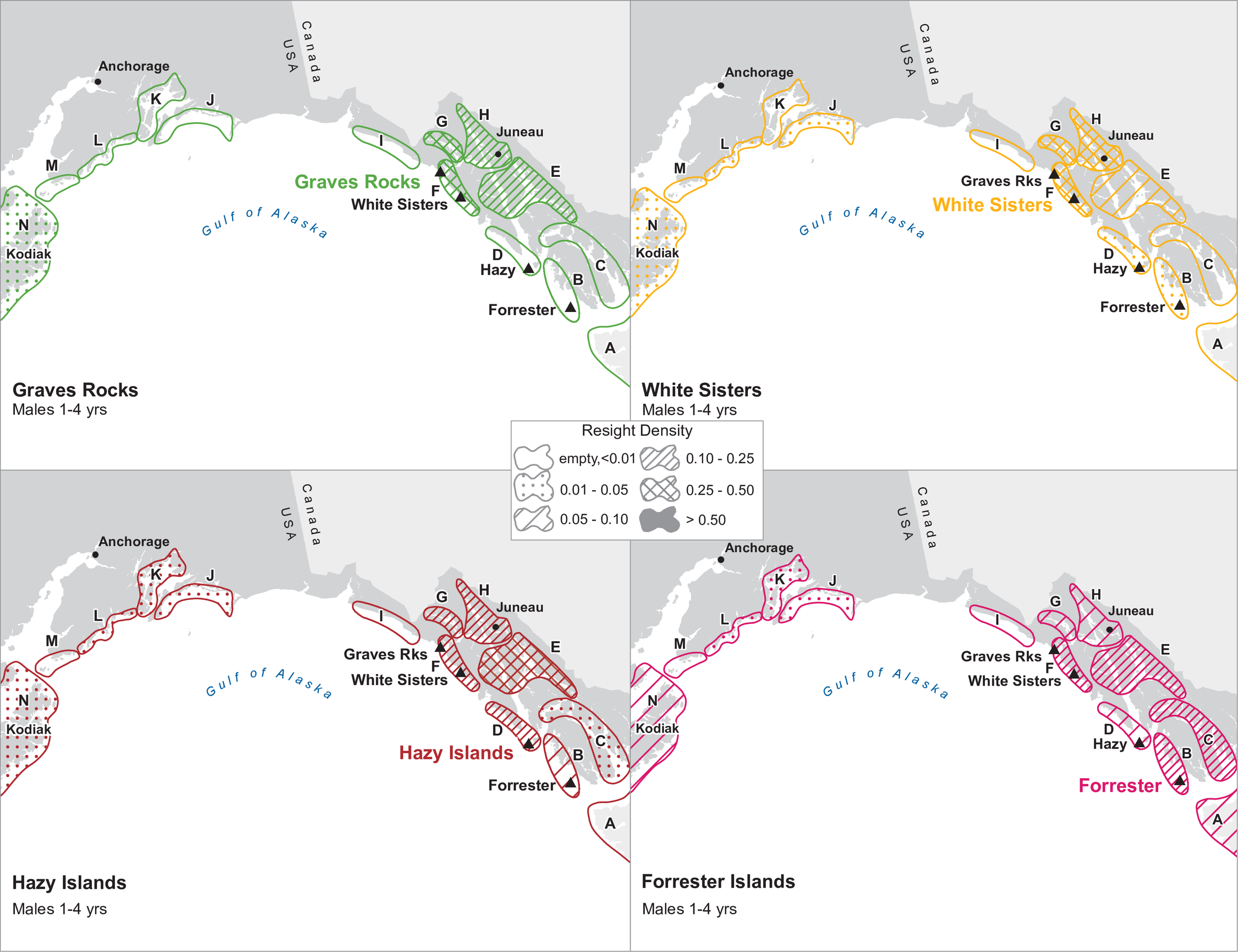

Supplement: S3 Fig — Breeding season distribution of juvenile male Steller sea lions born in the eastern stock based on an index of resight density. Resight density is the proportion of Steller sea lions seen within a region relative to the total number of sea lions from that natal rookery seen anywhere. Regions O, P, and Q are not included on eastern stock male maps; refer to Table 2 for densities of these areas. (TIF) [file pone.0208093.s005.tif]

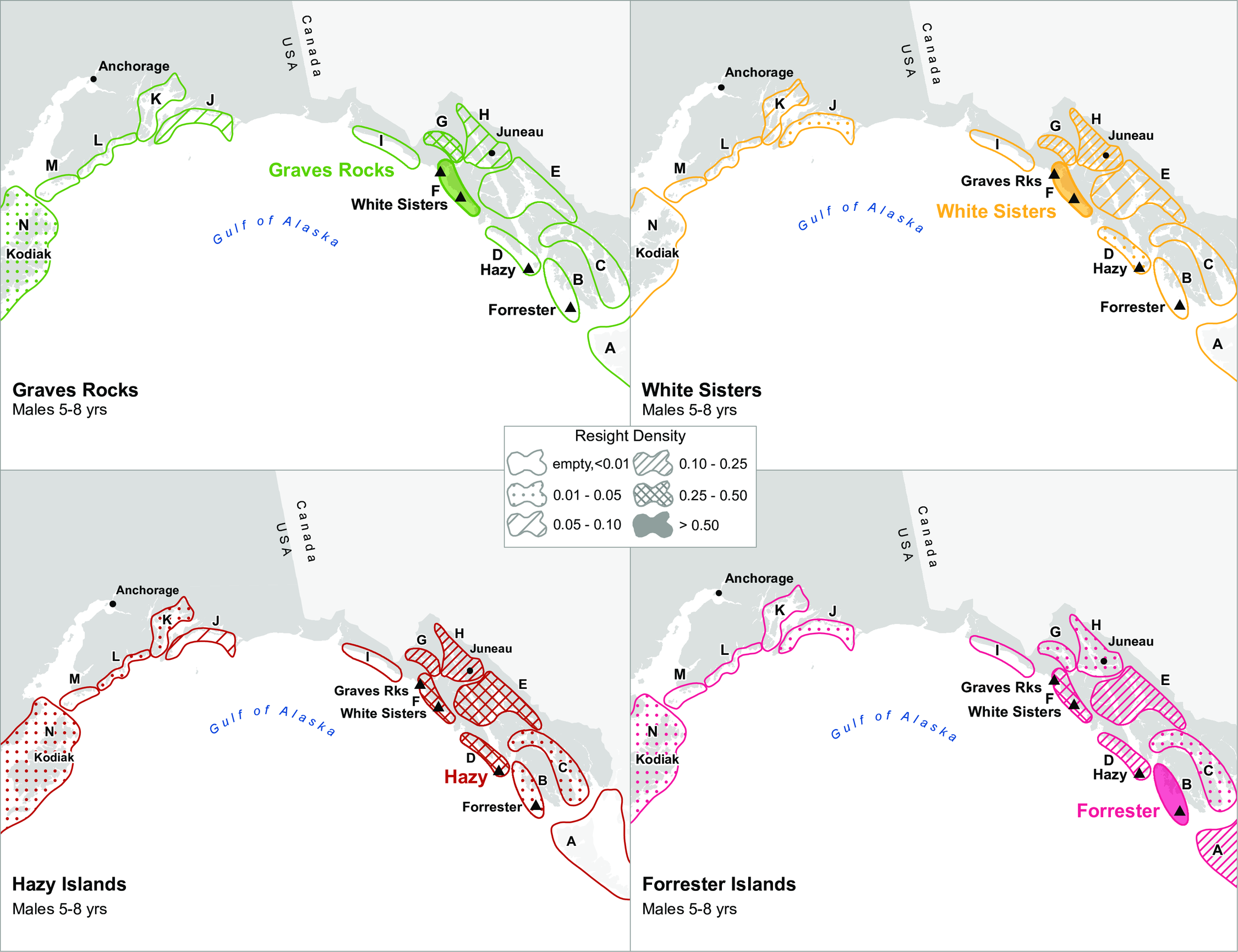

Supplement: S4 Fig — Breeding season distribution of sub-adult male Steller sea lions born in the eastern stock based on an index of resight density. Resight density is the proportion of Steller sea lions seen within a region relative to the total number of sea lions from that natal rookery seen anywhere. Regions O, P, and Q are not included on eastern stock male maps; refer to Table 2 for densities of these areas. (TIF) [file pone.0208093.s006.tif]

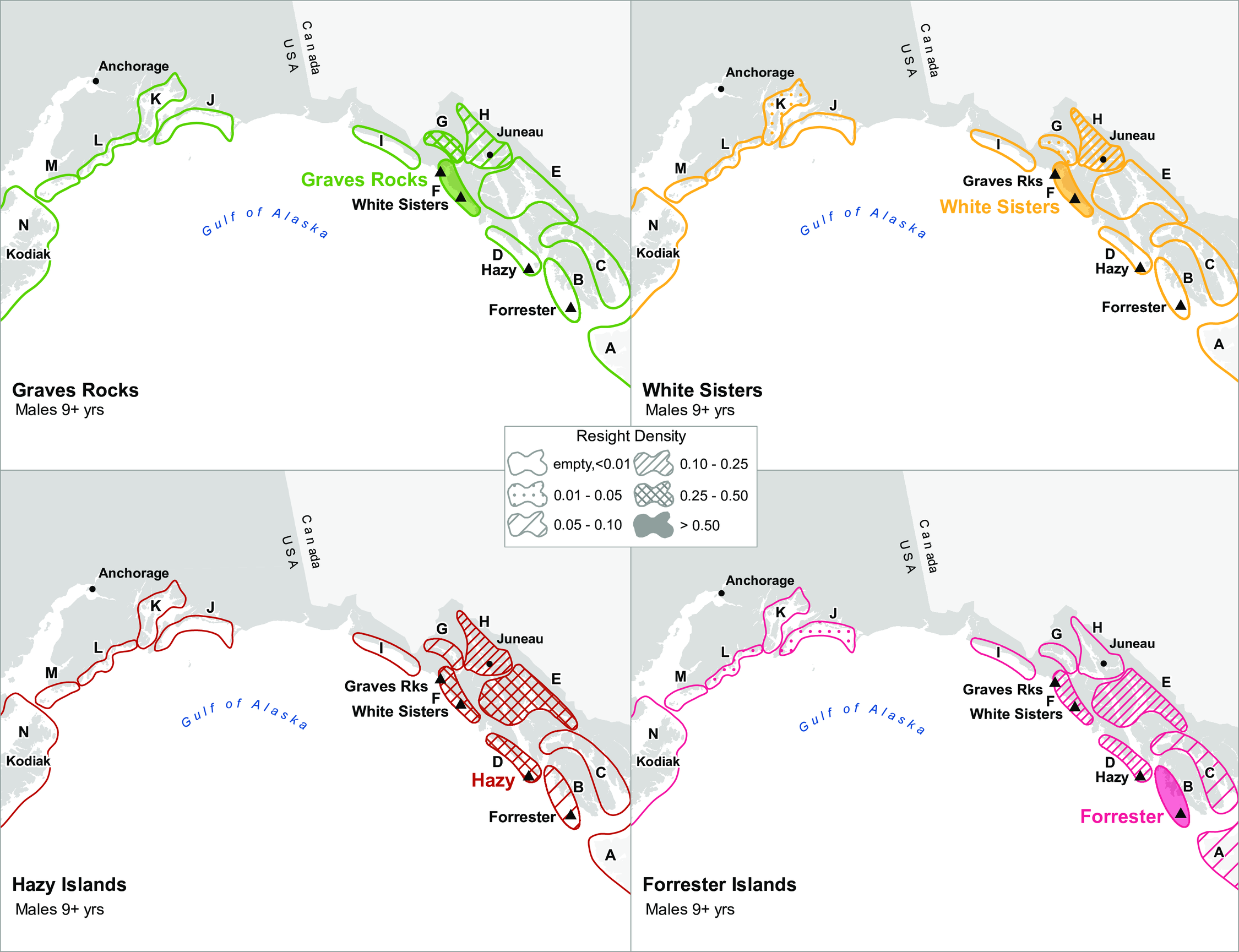

Supplement: S5 Fig — Breeding season distribution of adult male Steller sea lions born in the eastern stock based on an index of resight density. Resight density is the proportion of Steller sea lions seen within a region relative to the total number of sea lions from that natal rookery seen anywhere. Regions O, P, and Q are not included on eastern stock male maps; refer to Table 2 for densities of these areas. (TIF) [file pone.0208093.s007.tif]

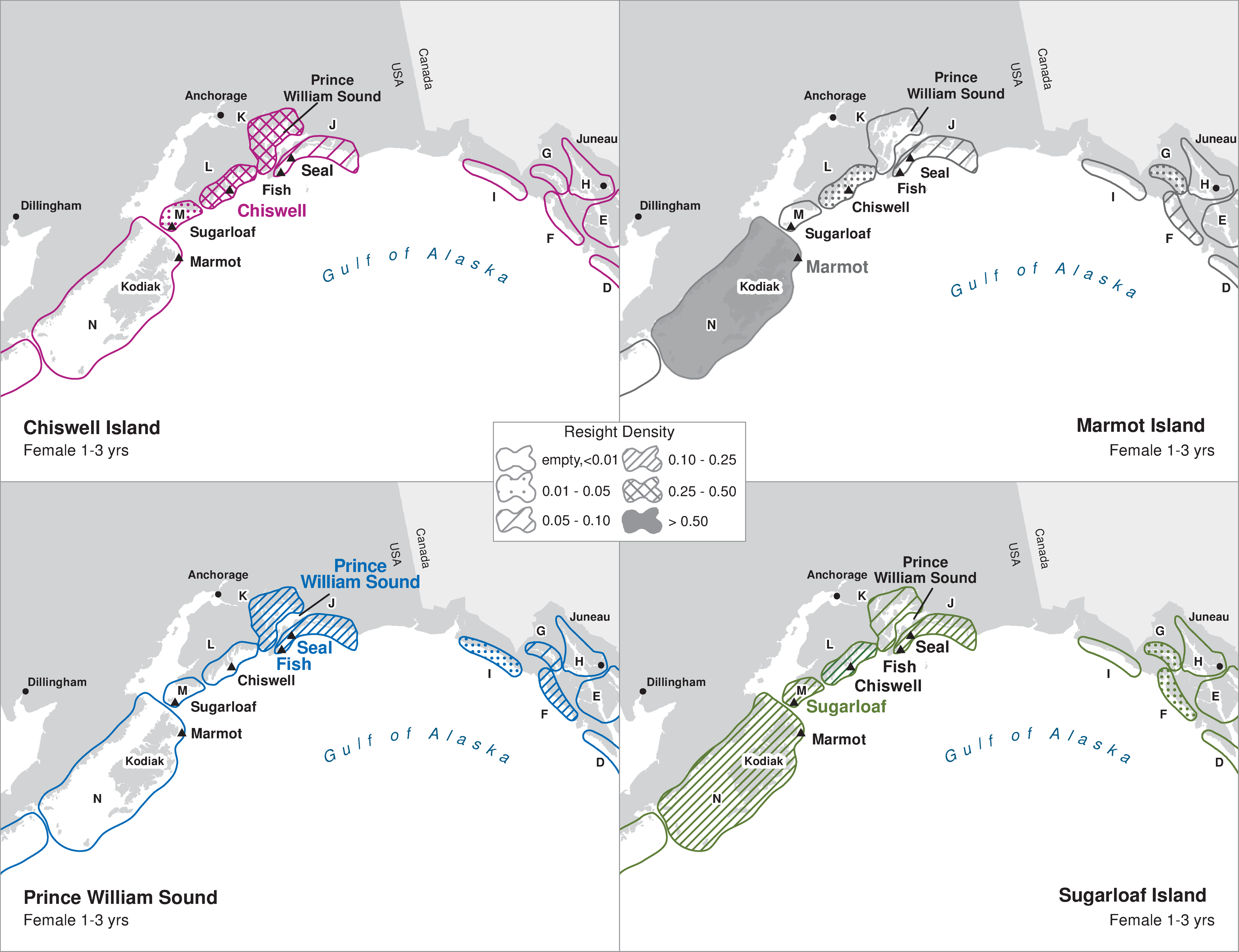

Supplement: S6 Fig — Breeding season distribution of juvenile female Steller sea lions born in the western stock based on an index of resight density. Resight density is the proportion of Steller sea lions seen within a region relative to the total number of sea lions from that natal rookery seen anywhere. Regions A through C and O through Q are not included on the western stock female maps; refer to Table 2 for densities. (TIF) [file pone.0208093.s008.tif]

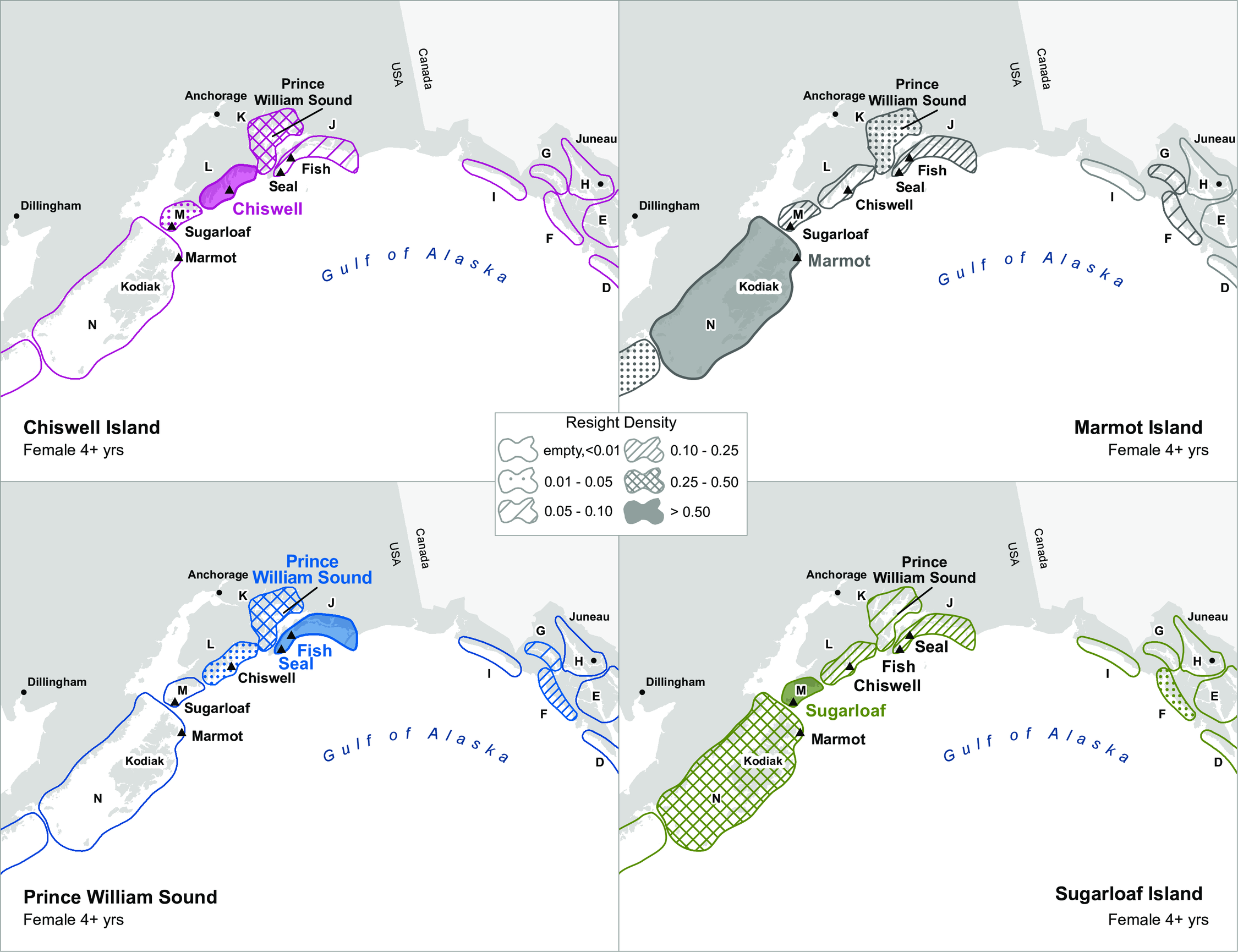

Supplement: S7 Fig — Breeding season distribution of adult female Steller sea lions born in the western stock based on an index of resight density. Resight density is the proportion of Steller sea lions seen within a region relative to the total number of sea lions from that natal rookery seen anywhere. Regions A through C and O through Q are not included on the western stock female maps; refer to Table 2 for densities. (TIF) [file pone.0208093.s009.tif]

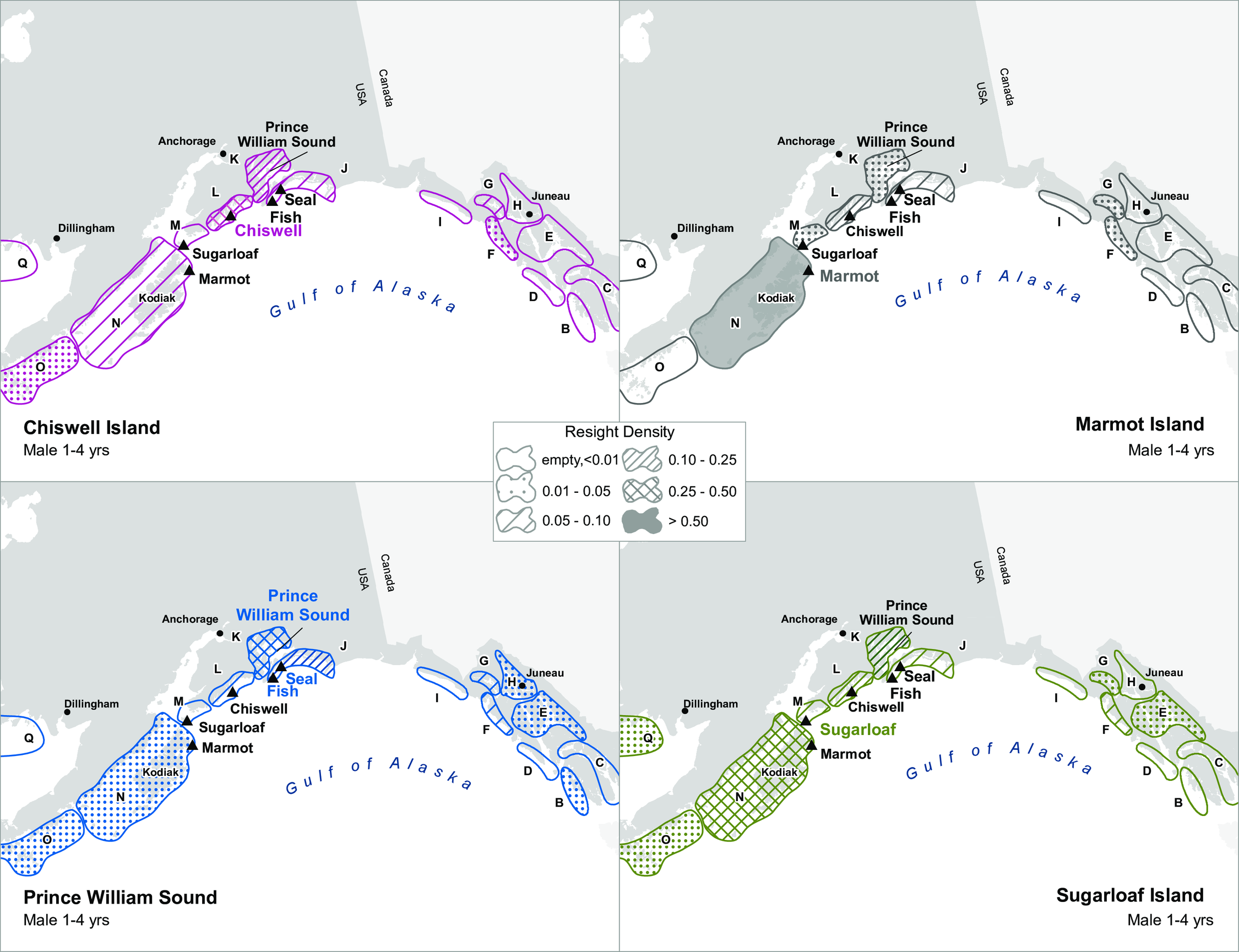

Supplement: S8 Fig — Breeding season distribution of juvenile male Steller sea lions born in the western stock based on an index of resight density. Resight density is the proportion of Steller sea lions seen within a region relative to the total number of sea lions from that natal rookery seen anywhere. Regions A and P are not included on western stock male maps; refer to Table 2 for densities. (TIF) [file pone.0208093.s010.tif]

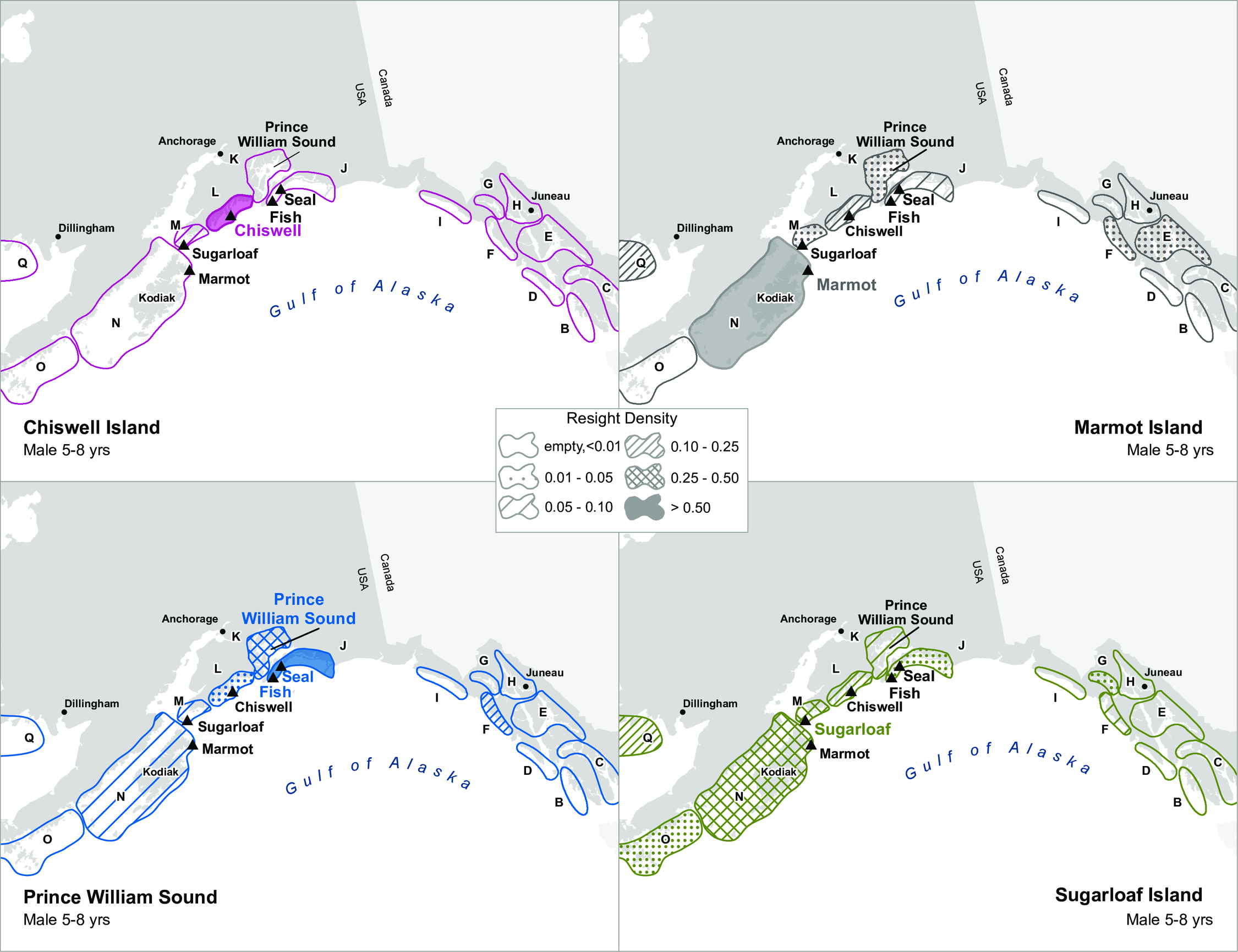

Supplement: S9 Fig — Breeding season distribution of sub-adult male Steller sea lions born in the western stock based on an index of resight density. Resight density is the proportion of Steller sea lions seen within a region relative to the total number of sea lions from that natal rookery seen anywhere. Regions A and P are not included on western stock male maps; refer to Table 2 for densities. (TIF) [file pone.0208093.s011.tif]

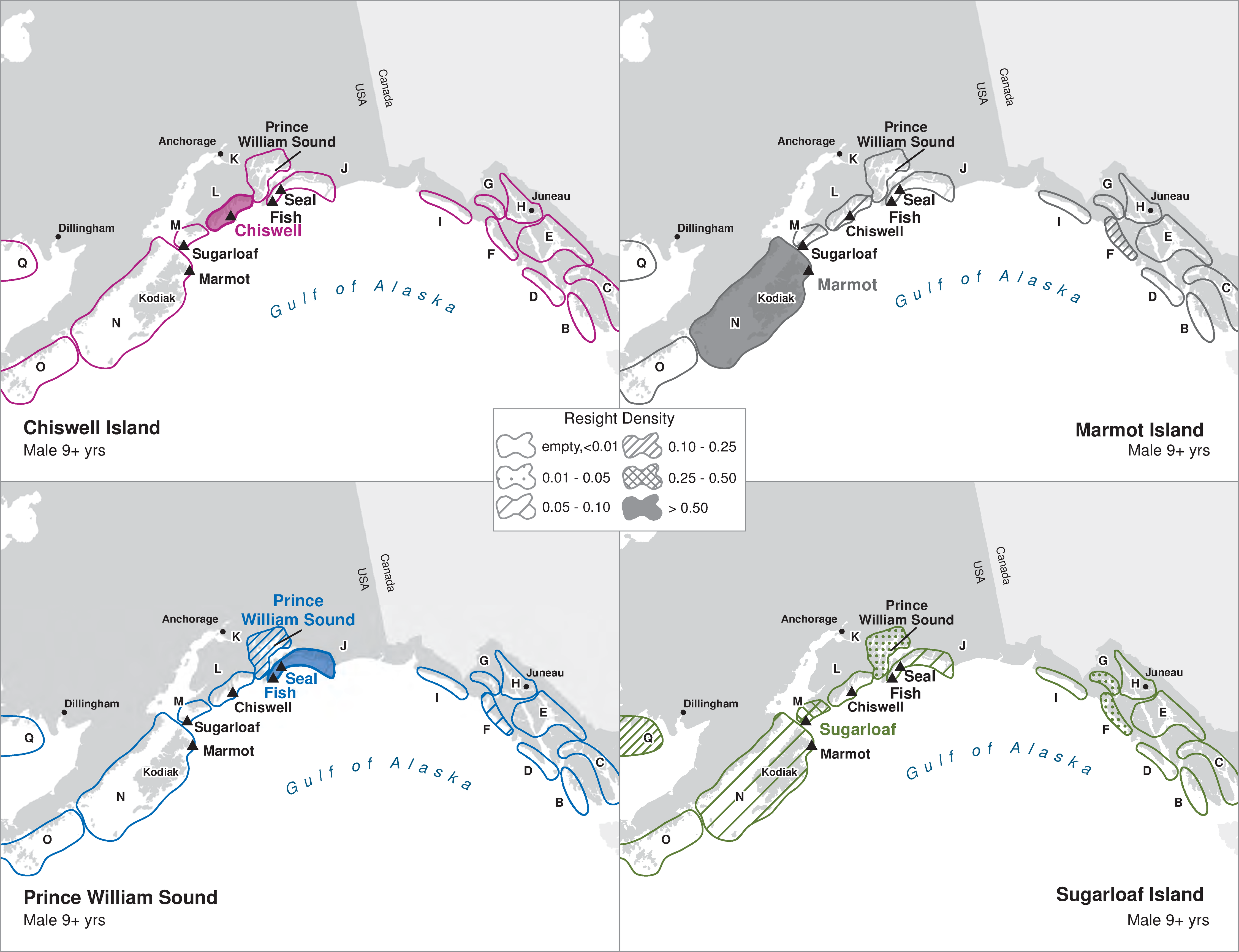

Supplement: S10 Fig — Breeding season distribution of adult male Steller sea lions born in the western stock based on an index of resight density. Resight density is the proportion of Steller sea lions seen within a region relative to the total number of sea lions from that natal rookery seen anywhere. Regions A and P are not included on western stock male maps; refer to Table 2 for densities. On map of Chiswell males age 9+, note that this represents only 3 individuals (all other maps represent ≥10 individuals). (TIF) [file pone.0208093.s012.tif]
