# Supplementary material for: Spatial distribution, movements, and geographic range of Steller sea lions (Eumetopias jubatus) in Alaska
Source: PLoS One. 2018 Dec 26;13(12):e0208093. doi: 10.1371/journal.pone.0208093 (PMC6306159; doi:10.1371/journal.pone.0208093)
Supplement: S1 Table — Values are mean distance (95% CI), maximum distance, and sample size. (DOCX) [file pone.0208093.s013.docx]

**S1 Table. Mean and maximum distance between Steller sea lion locations and natal rookery during the breeding season.** Values are mean distance, (95% confidence intervals), maximum distance (km), and sample size.

| Sex | Age | Forrester + Hazy | W. Sisters + Graves | PWS^a^ | Chiswell | Sugarloaf | Marmot | Ugamak |
| --- | --- | --- | --- | --- | --- | --- | --- | --- |
| F | 1 | 159  (144,172)  490, 273 | 76  (57,96)  292, 77 | 416  (242,590)  698, 14 | 67  (43,91)  280, 45 | 192  (142,243)  923, 61 | 180  (109,251)  1040,73 | 109  (77,141)  587, 62 |
|  | 2 | 182  (165,200)  585, 197 | 96  (78,115)  292, 78 | 281  (170,393)  698, 29 | 63  (34,92)  280, 36 | 259  (148,370)  947, 32 | 202  (112,291)  1016, 52 | 161  (93,228)  587, 45 |
|  | 3 | 143  (125,162)  1095, 213 | 84  (62,106)  751, 88 | 315  (150,479)  698, 16 | 50  (27,73)  206, 43 | 269  (181,357)  1001, 39 | 237  (134,340)  1119, 50 | 127  (83,171)  587, 76 |
|  | 4 | 83  (70,96)  435, 260 | 44  (29,59)  260, 85 | 257  (158,355)  698, 35 | 41  (18,64)  206, 41 | 148  (87,209)  923, 46 | 124  (52,195)  1016, 53 | 55  (23,87)  587, 93 |
|  | 5 | 46  (34,58)  429, 199 | 41  (26,55)  260, 88 | 214  (110,318)  698, 34 | 13  (0,29)  206, 34 | 122  (62,183)  991, 52 | 151  (76,226)  962, 51 | 47  (13,82)  587, 67 |
|  | 6 | 51  (38,63)  490, 200 | 39  (21,56)  355, 75 | 76  (20,131)  698, 44 | 19  (0,39)  206, 29 | 97  (49,144)  435, 39 | 148  (51,245)  1016, 39 | 42  (13,71)  587, 59 |
|  | 7 | 61  (45,78)  490, 157 | 59  (38,80)  355, 77 | 99  (34,164)  698, 41 | 32  (0,63)  173, 17 | 137  (52,222)  991, 39 | 102  (18,186)  1016, 41 | 35  (10,61)  587,55 |
|  | 8 | 66  (48,83)  490, 171 | 53  (24,81)  751, 63 | 112  (43,181)  698, 45 | 173  (--,--)  173,1 | 101  (30,172)  991, 42 | 155  (63,247)  1016, 41 | 63  (9,117)  587, 31 |
|  | 9 | 57  (39,74)  490, 164 | 45  (24,66)  355, 71 | 116  (39,194)  698, 34 | 173  (--,--)  173,1 | 66  (24,109)  435, 38 | 192  (67,316)  937, 25 | 75  (9,142)  587, 29 |
|  | 10 | 44  (27,61)  490, 141 | 47  (24,70)  260, 37 | 37  (0,82)  619, 28 |  | 119  (47,191)  991, 43 | 118  (13,224)  937, 26 | 210  (42,378)  587, 11 |
|  | 11 | 47  (29,65)  364, 109 | 39  (2,76)  355, 23 | 61  (0,146)  619, 21 |  | 29  (0,66)  336, 25 | 92  (15,170)  451, 17 | 195  (0,444)  587, 7 |
|  | 12 | 39  (16,62)  355, 56 | 24  (0,50)  219, 23 | 17  (0,39)  131, 13 |  | 87  (0,182)  923, 22 | 102  (29,175)  375, 17 |  |
|  | 13 | 17  (2,32)  224, 47 |  | 0  (0,0)  0, 6 |  | 58  (0,124)  336, 16 | 509  (0,5953)  937, 2 |  |
|  | 14 |  |  |  |  | 23  (0,72)  294, 13 | 254  (0,728)  937, 5 |  |
| M | 1 | 255  (221,288)  2270, 262 | 203  (142,263)  1955, 77 | 350  (150,550)  1992, 29 | 55  (29,80)  233, 37 | 249  (193,306)  1138, 91 | 156  (107,205)  994, 84 | 144  (114,174)  844, 109 |
|  | 2 | 354  (303,404)  1470, 152 | 145  (119,170)  834, 78 | 232  (120,343)  921, 26 | 205  (55,355)  847, 19 | 250  (177,323)  1194, 46 | 287  (169,404)  1280, 46 | 288  (206,370)  844, 42 |
|  | 3 | 438  (360,517)  2370, 117 | 201  (145,256)  1129, 66 | 287  (0,576)  921, 9 | 129  (46,213)  847, 35 | 447  (330,564)  1204, 46 | 408  (247,570)  1957, 37 | 286  (219,353)  587, 61 |
|  | 4 | 397  (323,470)  2836, 147 | 201  (119,283)  2260, 65 | 193  (83,303)  921, 25 | 156  (53,259)  846, 32 | 459  (326,593)  1194, 32 | 294  (93,495)  1633, 24 | 271  (198,344)  587, 61 |
|  | 5 | 336  (251,422)  2836, 108 | 128  (70,186)  2218, 87 | 168  (58,278)  645, 16 | 3  (0,6)  13, 16 | 249  (115,383)  947, 25 | 343  (101,585)  1633, 25 | 230  (170,290)  587, 84 |
|  | 6 | 185  (146, 224)  2270, 160 | 106  (56,156)  2260, 97 | 115  (46,184)  645, 31 | 14  (0,34)  162, 17 | 254  (74,434)  923, 11 | 389  (0,818)  1633, 13 | 97  (42,152)  587, 53 |
|  | 7 | 186  (144,227)  1054, 127 | 112  (44,180)  2260,79 | 45  (20,69)  131, 16 | 7  (0,19)  35, 7 | 225  (42,407)  947, 14 | 150  (15,286)  966, 24 | 54  (13,94)  587, 39 |
|  | 8 | 171  (129,213)  1033, 104 | 71  (42,100)  751, 77 | 81  (13,149)  645, 21 | 16  (0,60)  35, 3 | 203  (0,423)  1001, 10 | 224  (48,399)  1170, 22 | 122  (24,220)  587, 22 |
|  | 9 | 169  (132,207)  1204, 107 | 79  (18,140)  2260, 75 | 91  (0,197)  645, 17 | 12  (0,62)  35, 3 | 203  (16,390)  1001, 12 | 200  (0,450)  937, 11 | 132  (51,213)  587, 32 |
|  | 10 | 100  (67,133)  435, 72 | 60  (3,118)  751, 27 | 61  (0,136)  645, 18 |  | 207  (0,758)  1001, 5 | 234  (0,597)  937, 8 | 170  (8,332)  587, 10 |
|  | 11 | 118  (45,192)  1204, 40 | 24  (1,47)  81, 13 | 20  (0,74)  98, 5 |  | 368  (0,1109)  1001, 4 | 156  (0,558)  937, 6 | 137  (0,370)  587, 6 |
|  | 12 | 125  (50,201)  435, 18 | 34  (12,57)  81, 12 | 63  (0,156)  131, 4 |  | 331  (0,854)  1001, 6 | 247  (0,612)  971, 8 | 587  (--,--)  587, 1 |
|  | 13 | 283  (0, 570)  1204, 9 |  |  |  |  | 937  (--,--)  927, 1 |  |

^a^ PWS = Prince William Sound
